# Mindfulness-based interventions for children and adolescents with attention-deficit/hyperactivity disorder: a Bayesian meta-analysis of randomized controlled trials

**DOI:** 10.3389/fpsyg.2026.1711994

**Published:** 2026-03-11

**Authors:** Yiran Liu, Qiang Yan, Shiao Zhao, Sanfan Ng, Xianfei Wang, Ziheng Ning

**Affiliations:** 1 South China Business College of Guangdong University of Foreign Studies, Guangzhou, China; 2 Faculty of Health Sciences and Sports, Macao Polytechnic University, Macao, China; 3 College of Physical Education, Hainan Normal University, Haikou, China

**Keywords:** ADHD, adolescents, Bayesian meta-analysis, children, mindfulness-based interventions

## Abstract

**Background:**

Attention-deficit/hyperactivity disorder (ADHD) in children and adolescents is characterized by inattention, hyperactivity, and impulsivity, and is frequently accompanied by impairments in executive functioning, task performance, and emotion regulation. Mindfulness-based interventions (MBIs) have been increasingly evaluated as non-pharmacological approaches for ADHD, but findings remain heterogeneous.

**Objective:**

To synthesize evidence from randomized controlled trials (RCTs) on the effects of MBIs for youths with ADHD using a pre-registered Bayesian random-effects systematic review and meta-analysis, and to examine potential moderators (age) and dose–response relationships (contact hours).

**Methods:**

We conducted a pre-registered Bayesian random-effects systematic review and meta-analysis of RCTs evaluating MBIs in children and adolescents with ADHD. Seven databases were searched from inception to April 30, 2025, prioritizing immediate post-intervention outcomes. Seventeen RCTs (total *n* = 2,991) were included. Pooled effects were summarized as Hedges' g with 95% credible intervals (CrIs). Symptom-domain subgroup models were performed, and heterogeneity was quantified using I^2^ and τ^2^. Age-stratified analyses (mean age >10 years vs. ≤ 10 years) and dose–response modeling based on contact hours were conducted.

**Results:**

Across all outcomes, MBIs showed a small-to-moderate advantage over control conditions (Hedges' g = 0.49, 95% CrI 0.37–0.62), with substantial heterogeneity (I^2^ = 81.6%; τ^2^ = 0.16). Domain-specific subgroup models indicated statistically credible improvements in inattention (Hedges' g = 0.30, 95% CrI 0.12–0.50), hyperactivity/impulsivity (Hedges' g = 0.54, 95% CrI 0.31–0.78), executive functions (Hedges' g = 0.23, 95% CrI 0.05–0.43), global ADHD measures (Hedges' g = 1.23, 95% CrI 0.65–1.80), and task performance (Hedges' g = 0.37, 95% CrI 0.07–0.70). The estimate for emotion regulation was imprecise and included the null (Hedges' g = 0.42, 95% CrI −0.08–0.92). Age-stratified analyses suggested larger effects in samples with mean age >10 years than in those with mean age ≤ 10 years. Dose–response modeling suggested that higher contact hours may be associated with greater improvements in selected domains (notably hyperactivity/impulsivity), although uncertainty remained in several domains.

**Conclusion:**

MBIs may be a promising complementary approach for improving ADHD-related outcomes in youths. However, substantial heterogeneity and risk-of-bias considerations warrant cautious interpretation and underscore the need for larger, methodologically rigorous RCTs.

**Systematic review registration:**

https://www.crd.york.ac.uk/PROSPERO/view, Identifier: CRD420251079766 Public.

## 1 Introduction

Attention-deficit/hyperactivity disorder (ADHD) is a common neurodevelopmental condition in childhood and adolescence, characterized by developmentally inappropriate levels of inattention, hyperactivity, and impulsivity (Faraone et al., 2021; Polanczyk et al., 2007). Beyond these core symptoms, many affected youths experience broad functional impairments involving executive functioning, academic achievement, social relationships, and daily self-management (Faraone et al., 2021; Js et al., 2008; Loe and Feldman, 2007). These difficulties can persist across developmental stages and are frequently accompanied by emotional and behavioral problems, increasing the burden on families, schools, and healthcare systems (Faraone et al., 2006; Wolraich et al., 2019). Because symptom expression and associated impairments evolve with age, interventions for ADHD in youth must address both core symptom reduction and downstream functional outcomes (Faraone et al., 2006; Wolraich et al., 2019).

Pharmacological treatment remains a widely used first-line option and can be effective for many children and adolescents (Cortese et al., 2018; Wolraich et al., 2019). However, medication may be limited by side effects, adherence challenges, access barriers, caregiver preferences, and residual functional difficulties that are not fully resolved by symptom control alone (Cortese et al., 2018; Shaw et al., 2012; Wolraich et al., 2019). Consequently, there is sustained interest in evidence-based non-pharmacological approaches that can complement standard care, provide skills for self-regulation, and offer developmentally appropriate strategies for youths and their caregivers (Daley et al., 2014; Sonuga-Barke et al., 2013; Wolraich et al., 2019).

Mindfulness-based interventions (MBIs) have been increasingly tested as a non-pharmacological option for ADHD, with the general premise that systematic training in present-moment attention and self-regulation may help reduce inattentive and impulsive responding and support adaptive functioning (Evans et al., 2018; Lee et al., 2022; Oliva et al., 2021). In recent years, randomized controlled trials have evaluated MBIs across diverse settings and formats, including school-based programs, clinic-delivered interventions, and protocols involving caregivers (Meppelink et al., 2024; Siebelink et al., 2022; Valero et al., 2022; Wong et al., 2023). Reported outcomes extend beyond core symptom ratings to executive function, task performance, and emotion-related difficulties that are clinically salient in youth ADHD (Evans et al., 2018; Holopainen et al., 2025; Oliva et al., 2021).

Despite this growing literature, findings remain inconsistent, and it is not yet clear under which conditions MBIs yield reliable benefits for specific ADHD-related domains (Evans et al., 2018; Lee et al., 2022; Oliva et al., 2021). Apparent discrepancies likely reflect substantial clinical and methodological heterogeneity (Evans et al., 2018; Lee et al., 2022; Oliva et al., 2021). Clinically, trial samples differ in age distribution, baseline symptom severity and comorbidity profiles, medication status, and family context (Evans et al., 2018; Oliva et al., 2021). Intervention characteristics also vary widely, including program type and content, delivery format (child-only vs. family-involved), total contact hours, session frequency, instructor expertise, and adherence (Evans et al., 2018; Siebelink et al., 2022; Wong et al., 2023). Methodological variation further complicates synthesis, as control conditions range from waitlist and treatment-as-usual to active psychosocial comparators (Lee et al., 2022). Outcomes may be measured by informant ratings vs. performance-based tasks; assessment timing and reporting practices differ; and risk-of-bias features (such as limited blinding in subjective outcomes) may influence effect estimates (McGuinness and Higgins, 2021; Page et al., 2016; Sterne et al., 2019). Together, these sources of heterogeneity can obscure domain-specific effects and contribute to divergent conclusions across individual trials and prior syntheses.

To resolve these conflicting findings and address the substantial heterogeneity identified in previous trials, this study employed a Bayesian multi-level meta-analysis, while also examining symptom-specific outcomes. Based on existing literature and theoretical rationale, we propose the following hypotheses: (1) MBIs significantly reduce ADHD symptoms in children and adolescents; (2) The effectiveness of MBIs varies significantly across symptom domains, particularly in executive functioning, attention deficits, hyperactivity/impulsivity, emotional regulation, and overall functioning; and (3) Greater intervention dose, operationalized as total contact hours, would be associated with larger improvements in outcomes.

## 2 Methods

This systematic review and Bayesian meta-analysis was prospectively registered in PROSPERO (registration no. CRD420251079766) and conducted and reported in accordance with PRISMA 2020. Literature management, screening, and data extraction were supported by Covidence and ASReview; certainty of evidence was summarized using GRADEprofiler; and graphical outcome data were digitized using GetData Graph Digitizer. All statistical analyses were conducted in R (version 4.5.1), and key R packages and versions used for meta-analysis are reported in the Supplementary materials (R session information). The completed PRISMA 2020 checklist is available as Supplementary File S0.

### 2.1 Eligibility criteria

Eligibility criteria were formulated based on the PICOS framework.

**Participants:** Studies were included if participants were children and adolescents aged 6 to 18 years, formally diagnosed with ADHD according to the Diagnostic and Statistical Manual of Mental Disorders, Fifth Edition (DSM-5, American Psychiatric Association, 2013) or the International Classification of Diseases, Tenth Revision (ICD-10, World Health Organization, 2016).

**Interventions:** MBIs were defined following (Crane et al., 2017) and included structured programs such as Mindfulness-Based Stress Reduction (MBSR), Mindfulness-Based Cognitive Therapy (MBCT), Mindful Awareness Practices (MAPs), and MYmind. Studies were included only if mindfulness was delivered as a clearly distinguishable component.

**Comparators:** Eligible control groups included waitlist, treatment-as-usual (TAU), psychoeducation, or other non-mindfulness psychological interventions.

**Outcomes:** Primary outcomes included global ADHD ratings, inattention, hyperactivity/impulsivity, and executive function. Secondary outcomes comprised emotion regulation, and task performance. Specifically, executive function (EF) outcomes targeted higher-order cognitive processes and were assessed via two complementary approaches: (1) neurocognitive performance measures indexing core EF subcomponents, including inhibitory control, working memory, and cognitive flexibility; and (2) informant-rated measures of ecological executive functioning, primarily assessed using the Behavior Rating Inventory of Executive Function (BRIEF).

**Study design:** We considered randomized controlled trials (RCTs), including individually randomized trials and cluster-randomized controlled trials (cluster-RCTs).

**Exclusion criteria:** Studies were excluded if they were non-randomized controlled trials (non-RCTs), qualitative studies, or non-original articles (e.g., reviews, editorials). Additionally, studies were excluded if they did not report sufficient data for effect size computation, if mindfulness was not independently applied, or if the study was not published in English.

### 2.2 Information sources

A comprehensive search was performed across seven databases: PubMed, Cochrane Library, Embase, Scopus, Web of Science (WOS), ERIC, and EBSCOhost, covering the literature up to April 30, 2025. The search strategy combined Medical Subject Headings (MeSH) and free-text terms. In addition, the reference lists of included studies and relevant prior reviews were screened to identify potentially eligible trials not captured by database searches. The specific search strategies can be found in Supplementary File S8.

### 2.3 Study selection and data collection

**Figure 1:**
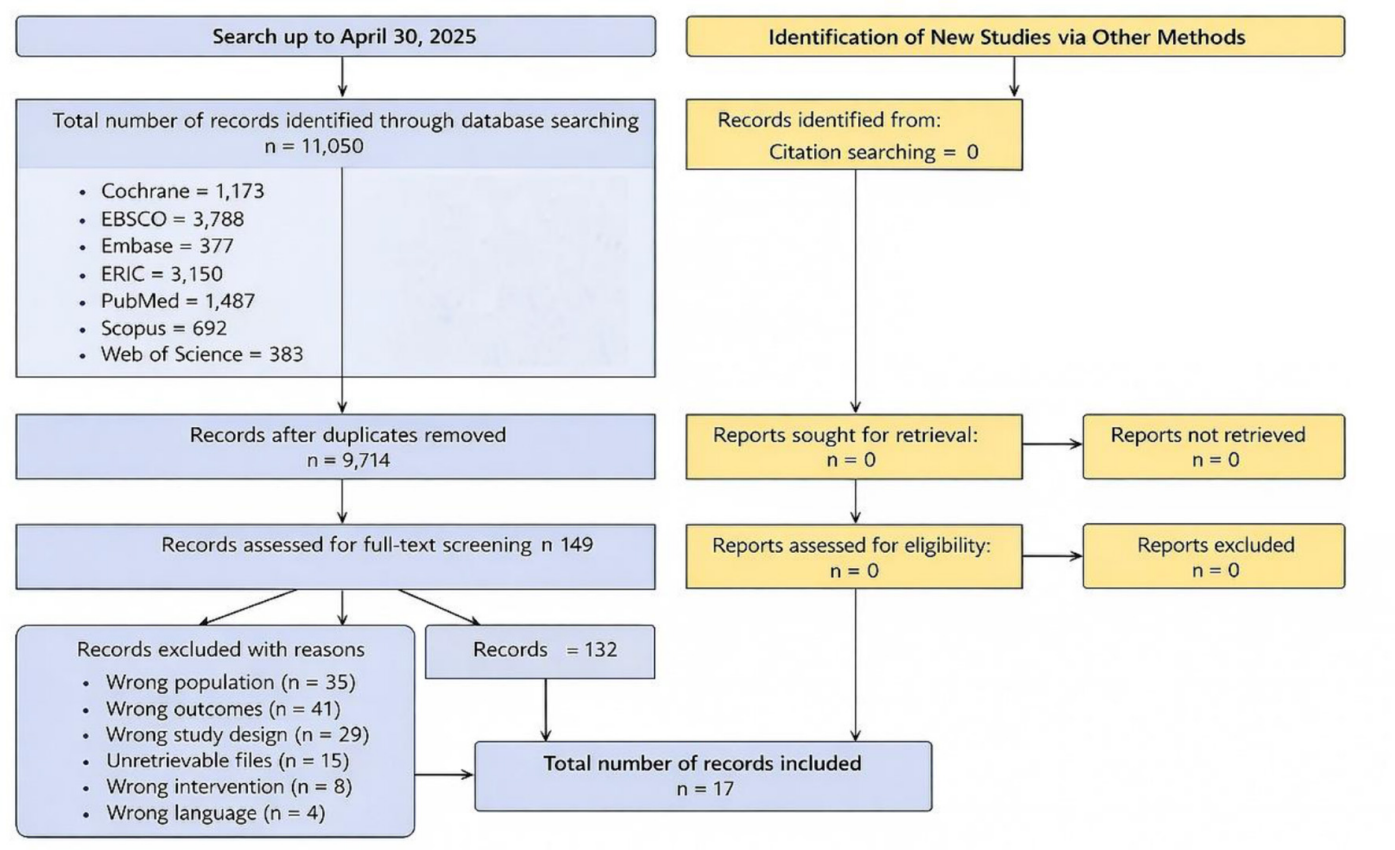
PRISMA flow diagram for study selection.

Title and abstract screenings were conducted using ASReview, an active learning-based artificial intelligence tool (van de Schoot et al., 2021). Prior to active learning, duplicates were removed and a small set of records was used to initialize the model based on reviewer-labeled relevant and irrelevant examples; ASReview then iteratively prioritized records predicted to be most likely relevant for manual screening. Screening was halted using the SAFE stopping rule after 200 consecutive records were judged irrelevant. Full-text screening and data extraction were conducted in Covidence using a piloted extraction form. Two reviewers (YL, QY) independently screened full texts and extracted data; discrepancies were resolved through discussion, and when needed, adjudicated by a third reviewer (ZN). The PRISMA flow diagram (Figure 1) summarizes the selection process. From 11,050 records, 280 duplicates were removed. After ASReview screening, 149 full-text articles were evaluated. Ultimately, 17 RCTs met the eligibility criteria and were included in the final synthesis.

### 2.4 Data items

The extracted data include the author, publication year, country/region, sample size, age and gender, intervention type and implementation form, instructor/implementation environment if reported, intervention frequency and duration, control conditions, outcome domains, assessment tools, and assessment time points. If multiple time points are reported, the outcome data immediately after the intervention are preferred. For each eligible comparison, the Hedges' g value (standardized mean difference adjusted for small samples) is used for effect size calculation. If the mean and standard deviation data are unavailable, the *t*-value or F-value is converted to the standardized mean difference according to the formula proposed by Lipsey and Wilson (2001). The GetData Graph Digitizer software (GetData, 2020) is used to digitize the graphical outcome data. When necessary, the Meta-Analysis Accelerator software (Abbas et al., 2024) is used to convert the summary data based on the median or range to the mean ± standard deviation.


$$\begin{array}{l} d=\sqrt{F\times \left(\frac{1}{n_{1}}+\frac{1}{n_{2}}\right)}\;d=t\times \sqrt{\left(\frac{1}{n_{1}}+\frac{1}{n_{2}}\right)} \end{array}$$

### 2.5 Risk of bias assessment

The risk of bias across all included randomized controlled trials was assessed in accordance with the Cochrane Handbook for Systematic Reviews of Interventions. Two reviewers (YL, QY) independently conducted the assessments using the Cochrane risk of bias (RoB2) tool embedded in the Covidence platform (McGuinness and Higgins, 2021). The tool evaluates seven key domains: (1) sequence generation, (2) allocation concealment, (3) blinding of participants and personnel, (4) blinding of outcome assessment, (5) incomplete outcome data, (6) selective outcome reporting, and (7) other potential biases. Each domain was rated as “low risk,” “some concerns,” or “high risk.” Discrepancies between reviewers were resolved through discussion, and all final decisions were documented. Studies exhibiting more than two but fewer than four areas designated as unclear risk were categorized as having moderate overall risk. The final risk profiles were visualized using the robvis package in R, and the results were summarized in both tabular and graphical formats.

### 2.6 Publication bias

Publication bias was examined using two approaches in the metafor package (Viechtbauer, 2010). First, Egger's regression test was conducted using the regtest() function (model = “lm”) to evaluate small-study effects. The test regressed the effect sizes on their standard errors. Since the power of Egger's test is low when the number of studies is small, *p* < 0.10 is conventionally used as the threshold for indicating asymmetry. Second, the trim-and-fill method was implemented using the function to estimate the number of potentially missing studies due to publication bias, and it was applied only when Egger's test indicated asymmetry (*p* < 0.1). The adjusted pooled effect sizes were calculated accordingly. Trim-and-fill funnel plots were exported and visually inspected. In addition, funnel plots were generated using the “viz_sunset()” function from the metaviz package, which integrates conventional funnel plot information with study-level statistical power. This power-enhanced visualization displays effect sizes against their standard errors while employing color gradients to indicate the statistical power of individual studies. By incorporating both significance contours and power contours, the sunset funnel plot facilitates a more nuanced evaluation of small-study effects and potential publication bias. The use of “viz_sunsets()” thus provides a more intuitive and informative graphical representation, enabling a clearer assessment of the robustness of the synthesized evidence and the influence of potential reporting bias.

### 2.7 Certainty in evidence

The quality of evidence regarding ADHD symptom outcomes was evaluated using the GRADE framework. The complete evidence profiles are provided in Supplementary file S9. Potential downgrades were considered across five domains:

**Risk of bias:** Certainty was not reduced when at least two-thirds of the cumulative information derived from studies judged at low risk of bias. When the preponderance of evidence came from studies with unclear/some concerns risk, we downgraded one level; when it chiefly came from high-risk studies, we downgraded two levels.

**Inconsistency:** We downgraded one level for substantial heterogeneity (I^2^ 50%−75%) and two levels for considerable heterogeneity (I^2^ > 75%).

**Imprecision:** Downgrading was applied if the 95% confidence interval crossed the line of no effect (e.g., 0 for standardized mean differences) and/or if the optimal information size was not achieved (for continuous outcomes, total sample size < 400). Meeting one of these criteria triggered a one-level downgrade; satisfying both led to a two-level downgrade.

**Indirectness:** We reduced certainty when important departures from the review question existed with respect to population, intervention, comparator, or outcome.

**Publication bias:** A one-level downgrade was applied when Egger's test indicated significant asymmetry and funnel plots showed consistent small-study effects.

### 2.8 Statistical analysis

Bayesian random-effects meta-analyses were conducted in R (version 4.5.1) using the bmeta and bayesmeta packages. Effect sizes were expressed as Hedges'g. Between-study heterogeneity was quantified by τ (tau). A weakly informative prior with a scale of 0.5 was placed on tau. Posterior inference was summarized by posterior means and 95% equal-tailed credible intervals (CrI), and heterogeneity was reported from the posterior distribution of tau.

Between-study heterogeneity was assessed with the I^2^ statistic, which quantifies the proportion of total variability attributable to true heterogeneity rather than sampling error. In the frequentist analyses, random-effects models were fitted using the metafor package in R (Viechtbauer, 2010) with the same specification used for pooling. From these models, Cochran's Q, τ^2^, and I^2^ were derived, and subgroup-specific I^2^ values were calculated similarly within each symptom domain. For the Bayesian models, heterogeneity was summarized by the posterior distribution of τ^2^ (reported as the posterior median with 95% credible intervals). To facilitate comparability with conventional meta-analytic summaries, we also report I^2^ from the frequentist framework. Following common conventions, I^2^ values near 25%, 50%, and 75% were interpreted as low, moderate, and high heterogeneity, respectively.


$$\begin{array}{l} Q=\overset{k}{\underset{i=1}{\sum }}w_{i}{\left(y_{i}-{\hat{\mu }}_{FE}\right)}^{2}\;df=k-1\\ I^{2}=\max\left\{0,\frac{Q-df}{Q}\right\}\times 100\% \end{array}$$

For the heterogeneity parameter τ^2^, a half-Cauchy prior (0, 0.5) was specified. This choice is supported by previous research, which suggests that weakly informative priors are appropriate for modeling heterogeneity in Bayesian random-effects meta-analysis (Röver et al., 2021). Models were fitted using Markov Chain Monte Carlo (MCMC) sampling, and convergence was assessed using Rhat values. The closer the Rhat value is to 1, the stronger the indication that the Markov chain has successfully converged to the target distribution (Gelman et al., 2013).

Posterior distributions were summarized using posterior means and 95% equal-tailed credible intervals (CrI). If the CrI excludes zero, this indicates evidence for a non-zero effect. Bayes factors were used to assess the strength of evidence for the effect, with values greater than 1 favoring the alternative hypothesis. The posterior distributions were evaluated using Markov Chain Monte Carlo (MCMC) methods, with Bayesian inference used to estimate the parameters. The overall findings were robust, with Bayes factors and credible intervals providing complementary evidence for the presence of a non-zero effect.

To evaluate the robustness of the pooled effects, we conducted a leave-one-out (LOO) sensitivity analysis in which each study was sequentially omitted and the random-effects meta-analysis was re-fitted under the same weighting scheme and between-study variance estimator as the primary model. The resulting series of pooled standardized mean differences (SMDs) with 95% confidence intervals were summarized in a LOO forest plot. Studies were considered potentially influential if their omission shifted the pooled SMD outside the 95% CI of the primary estimate. LOO analyses were implemented in R using the metafor package, and results were inspected for substantive deviations from the primary pooled effect.

### 2.9 Moderation analysis by dosage

Moderation analysis by dosage was performed using the metafor and mgcv packages in R. Dose was defined as total contact hours of the intervention. Study-level effects were computed as Hedges' g using the two-group standardized mean difference formula with small sample correction and corresponding sampling variances using the metafor package.

We fitted a weighted generalized additive model (GAM) with a thin-plate spline for total hours, estimated by REML with inverse-variance weights (1/vi) under a Gaussian identity link. Pointwise 95% confidence bands were derived from model-based standard errors, 80% and 60% bands were added for visual context. Extrapolation beyond the observed hour range was avoided.

The same weighted framework was applied within each pre-specified domain (Inattention, Hyperactivity/Impulsivity, Executive Functions, Global symptoms, Task Performance, and Emotion Regulation). When the available dose levels were insufficient to support a stable spline (fewer than 3 unique dose levels), we implemented a fallback approach: first attempting a weighted linear model or, if only one dose level was available, an intercept-only model, both using inverse-variance weights.

## 3 Results

The results are presented in seven parts: study selection, characteristics of the included studies, quality assessment, meta-analysis, subgroup analysis, sensitivity analysis, and publication bias.

### 3.1 Study selection

A total of 11,050 articles were initially identified through comprehensive searches across seven databases. After removing 280 duplicates, 10,770 records remained for title and abstract screening using ASReview. This step excluded 10,621 records based on irrelevance, leaving 149 full-text articles for further assessment. Following full-text screening, 132 articles were excluded based on pre-defined criteria. Ultimately, 17 randomized controlled trials (RCTs) met all eligibility criteria and were included in the final Bayesian meta-analysis (Figure 1: PRISMA Flow Chart).

### 3.2 Characteristics of the included studies

Seventeen studies comprising 2,991 participants were included. Trials were conducted between 2015 and 2025 across Europe (*n* = 9, 52.9%), Asia (*n* = 6, 35.3%), Africa (*n* = 1, 5.9%), and North America (*n* = 1, 5.9%), providing broad geographic coverage. Intervention content predominantly consisted of mindfulness programs (*n* = 16, 94.1%); one trial (5.9%) evaluated mindfulness combined with a behavioral treatment. Control conditions most commonly used waitlist comparators (*n* = 9, 52.9%), with fewer studies employing CBT-based protocols (*n* = 2, 11.8%) or psychoeducation (*n* = 2, 11.8%); single studies used an active non-CBT control, exercise, placebo, or pharmacologic comparators (each *n* = 1, 5.9%). When reported, programs had a median duration of 8 weeks (IQR ≈ 8–8.5; range 4–12) and a median total contact time of ~10 h (IQR ≈ 6.3–12; range 0.16–24). Outcomes most frequently targeted inattention and hyperactivity–impulsivity, with additional assessments of executive function, emotion regulation, and global functioning, typically measured using validated rating scales and or performance-based tasks. A detailed summary of study characteristics is provided in Supplementary file S1.

### 3.3 Quality assessment

**Figure 2:**
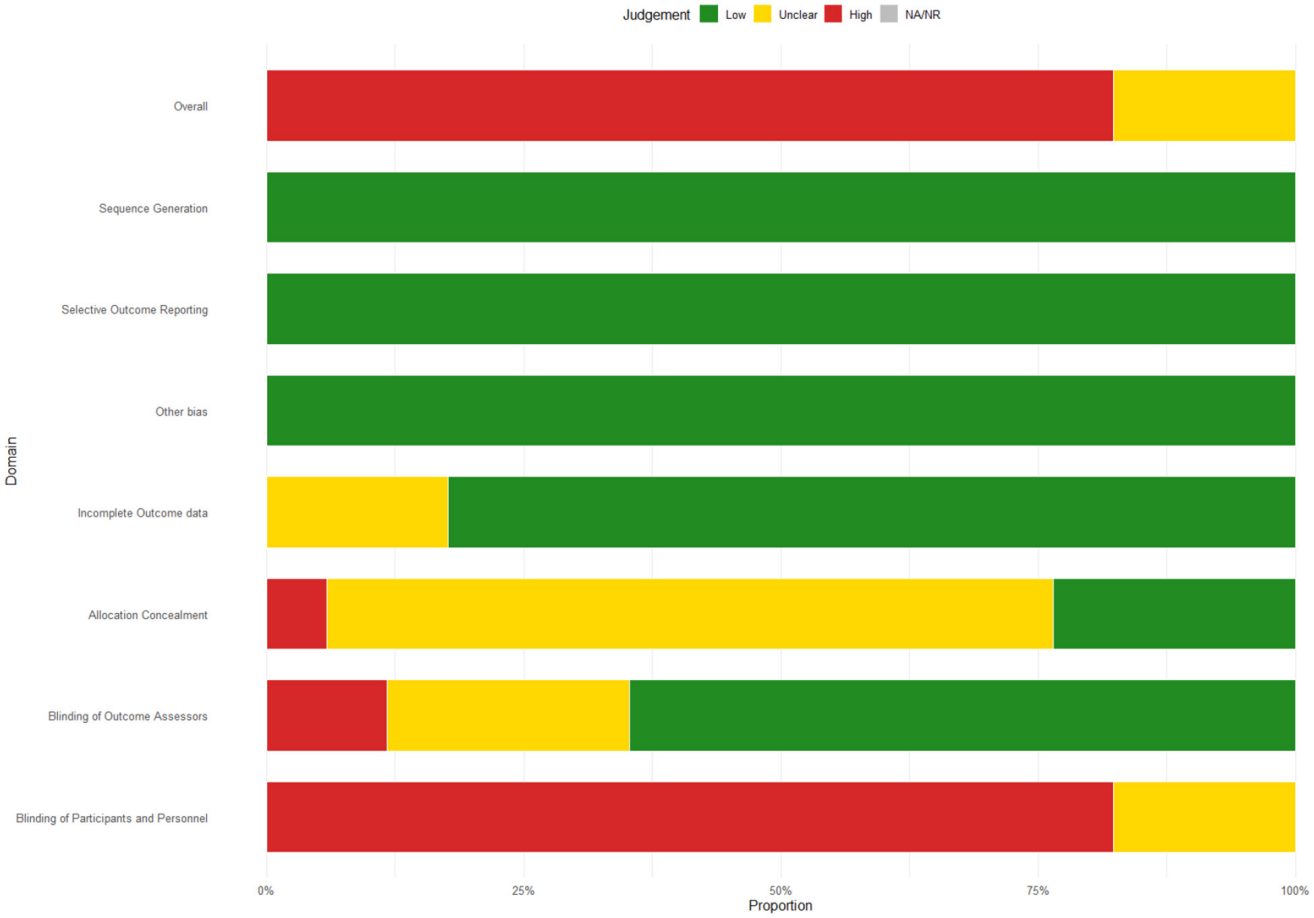
Risk of bias summary.

Bias from the randomization process was generally low or with some concerns, except for one trial (Lo et al., 2020) judged high risk due to quasi-random allocation methods (5.9%). For missing outcome data, most studies were low risk; however, one trial (Holopainen et al., 2025) showed high risk owing to substantial attrition (5.9%). Measurement of outcomes was the most affected domain, with six studies (Behbahani et al., 2017; Franco et al., 2016; Holopainen et al., 2025; Lo et al., 2020; Ponomarev et al., 2023; Putriana et al., 2025) rated high risk. These relied on subjective assessments without adequate blinding, leading to potential detection bias (35.3%). Selective reporting was not a major issue. Overall, more than 75% of the studies were classified as high risk, which undermines the robustness of the data results. Detailed study-level decisions and justifications are available in Supplementary file S2. The overall risk-of-bias summary is shown in Figure 2.

### 3.4 Meta-analysis

The Bayesian meta-analysis first estimated an overall effect model, which synthesized evidence across all included studies to determine the general effectiveness of mindfulness-based interventions on ADHD symptoms. MCMC diagnostics supported satisfactory convergence ($\hat{\text{R}}$ ≈ 1.00–1.01 across key parameters; effective sample sizes ≈ 2,000–3,000).

#### 3.4.1 Overall effect model

**Figure 3:**
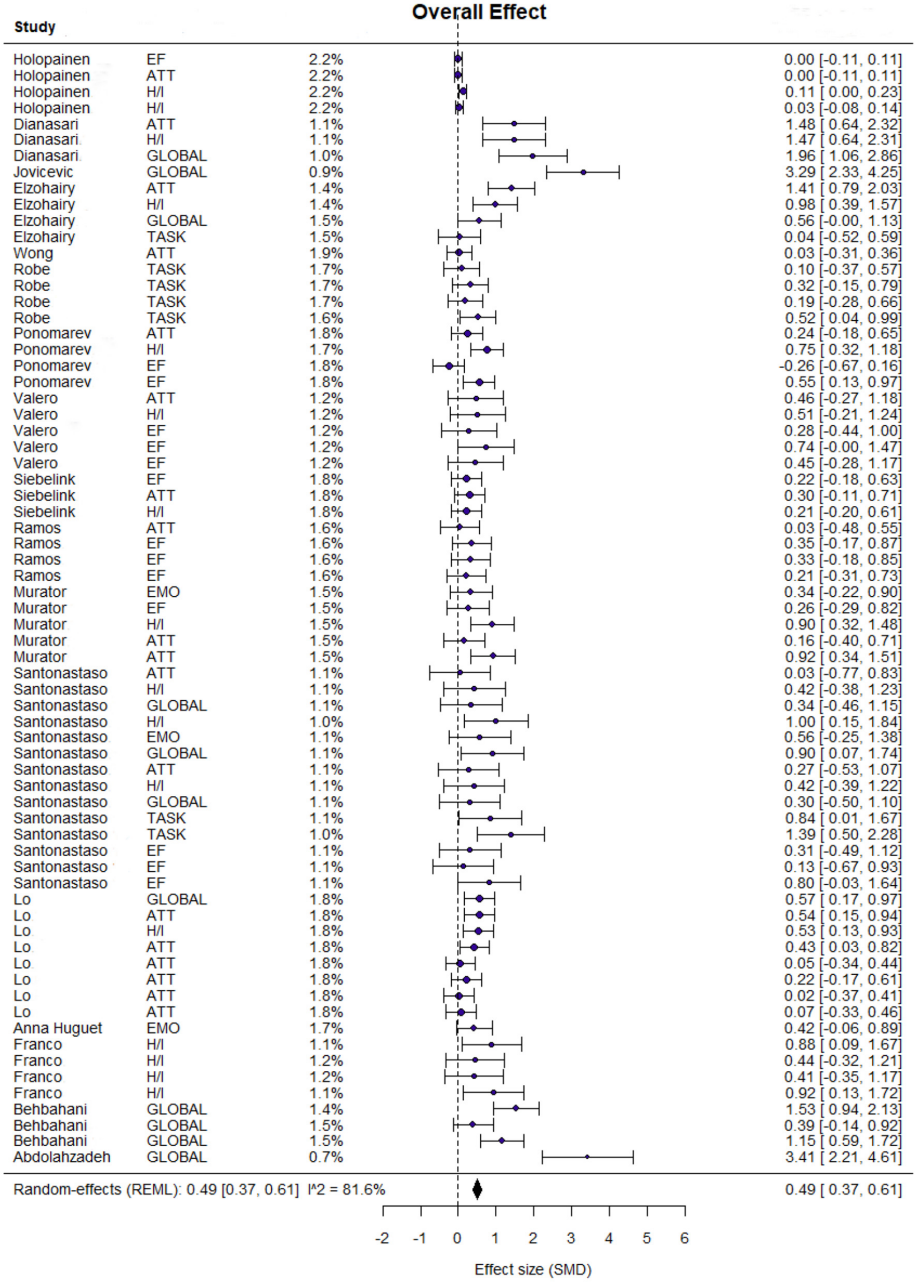
Forest plot of MBIs vs. control conditions on ADHD symptoms.

The Bayesian meta-analysis showed a statistically significant effect (Hedges' g) of 0.49 (95% credible interval [CrI]: 0.37 to 0.62), with moderate-to-high heterogeneity (I^2^ = 81.6%; τ^2^ = 0.16). A summary of the overall effect is presented in Figure 3.

**Figure 4:**
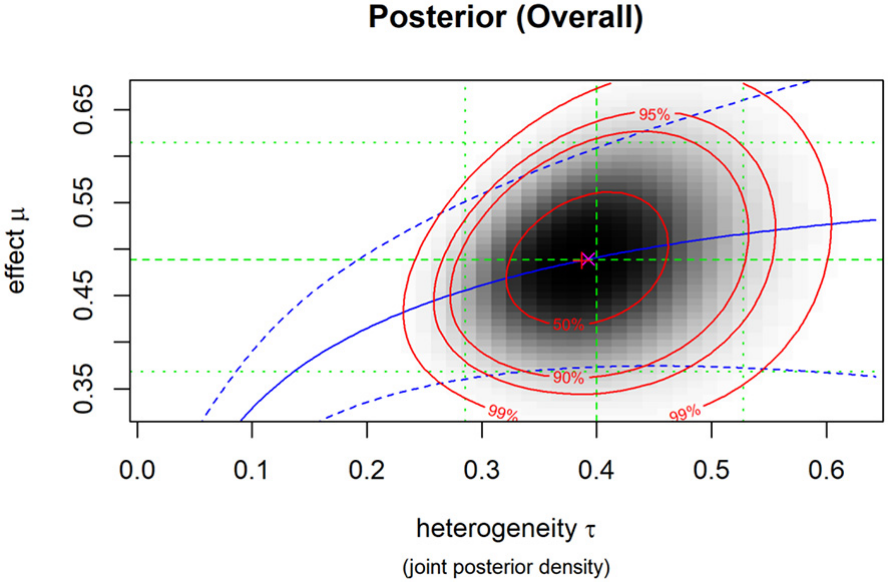
Posterior density plot of the overall effect.

The posterior mean was 0.49 (95% CrI, 0.37–0.62), indicating a small-to-moderate advantage of MBIs over control conditions on ADHD symptoms. The posterior median τ was 0.40, consistent with substantial heterogeneity. For comparison, the frequentist random-effects model yielded I^2^ ≈ 81.6% for the same dataset. The tilted credible contours indicate positive posterior dependence between μ and τ, such that larger heterogeneity is associated with wider uncertainty and slightly higher compatible values of μ. Given this substantial heterogeneity, the pooled estimate should be interpreted with caution, as it represents an average across studies with notable clinical and methodological differences. Specifically, variations in participant age (children vs. adolescents), intervention dosage (contact hours), and control conditions (active vs. waitlist) likely contribute to the observed variance. Consequently, the overall effect size may not precisely reflect the outcome for every specific clinical setting. The posterior density plot of the total effect is shown in Figure 4.

#### 3.4.2 Subgroup analyses by symptom domains

A statistically significant effect was observed in inattention (ATT) [μ = 0.3; 95% CrI 0.12–0.5], hyperactivity/impulsivity (H/I) [μ = 0.54; 95% CrI 0.31–0.78], executive functions (EF) [μ = 0.23; 95% CrI 0.05–0.43], global measures (GLOBAL) [μ = 1.23; 95% CrI 0.65–1.8], and task performance (TASK) [μ = 0.37; 95% CrI 0.07–0.7]. Emotion regulation did not show a statistically credible improvement (EMO: μ = 0.42; 95% CrI −0.08 to 0.92). The estimate was imprecise and the CrI included the null; therefore, evidence in this domain should be considered inconclusive.

**Figure 5:**
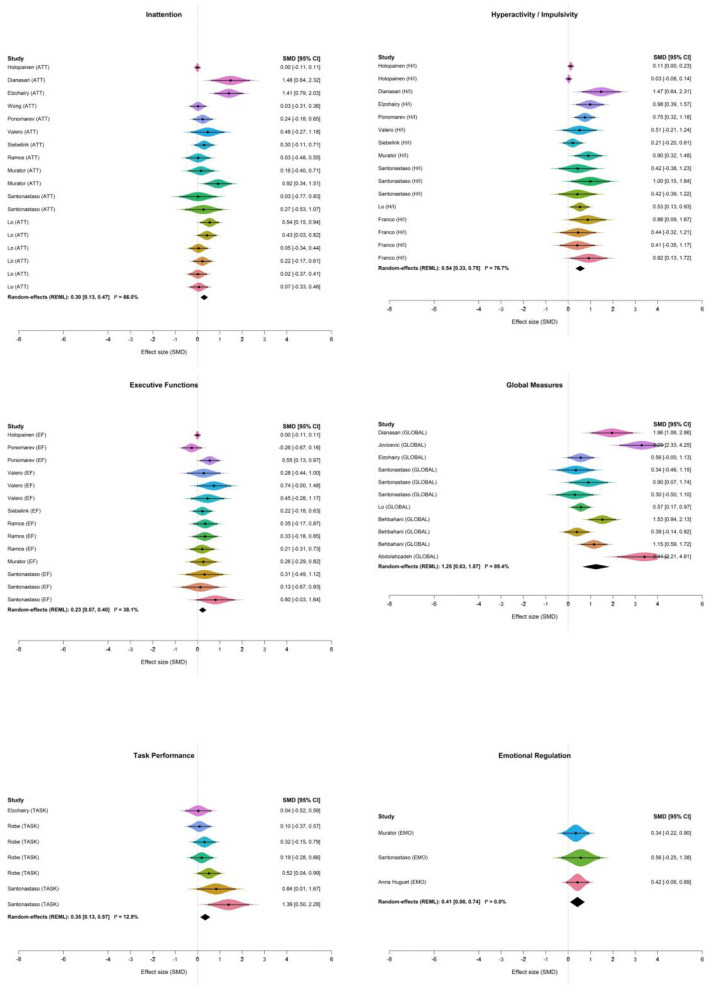
Forest plots by symptom domain (EF, H/I, ATT, GLOBAL, TASK, and EMO).

Heterogeneity estimates varied across subgroups. The GLOBAL subgroup exhibited substantial heterogeneity, with a posterior mean τ = 0.855 (I^2^ = 82.3%). In contrast, the ATT and H/I subgroups demonstrated moderate heterogeneity, with τ = 0.290 (I^2^ = 52.7%) and τ = 0.334 (I^2^ = 50.0%), respectively. The remaining subgroups EF, EMO, and TASK showed low to moderate heterogeneity, with τ = 0.196 (I^2^ = 28.5%), τ = 0.237 (I^2^ = 35.0%), and τ = 0.235 (I^2^ = 35.7%), respectively. The subgroup forest plot based on symptom grouping is shown in Figure 5.

Residual heterogeneity persisted after symptom-based subgrouping, indicating that effects likely vary across clinical and design features. Plausible clinical drivers include differences in participant age range, baseline symptom severity/comorbidity profiles, and intervention dose, format, and setting. Methodological contributors may include heterogeneity in control conditions, outcome operationalization (informant ratings vs. performance tasks), assessment timing, and risk-of-bias features (e.g., lack of blinding). Accordingly, pooled effects should be interpreted as average effects rather than a single precise estimate applicable to all contexts.

#### 3.4.3 Subgroup analyses by population

We employed a prespecified cut-off of 10 years to delineate the developmental transition from childhood to early adolescence and the shift to secondary education. Age subgroup analyses ( ≤ 10 vs. >10 years) were conducted to reflect developmental trajectories in executive functions—specifically attention and self-regulation—that may plausibly moderate the efficacy of MBIs (Best and Miller, 2010; Casey et al., 2008; Diamond, 2013). Age-stratified analyses indicated that children ( ≤ 10 years; k = 43) showed a pooled standardized mean difference of μ = 0.46 [95% CrI: 0.34, 0.59], while adolescents (>10 years; k = 26) demonstrated a larger pooled effect μ = 0.60 [95% CrI: 0.32, 0.92]. Although the credible intervals overlapped, the results suggest relatively stronger benefits of MBIs among adolescents than in younger children.

**Figure 6:**
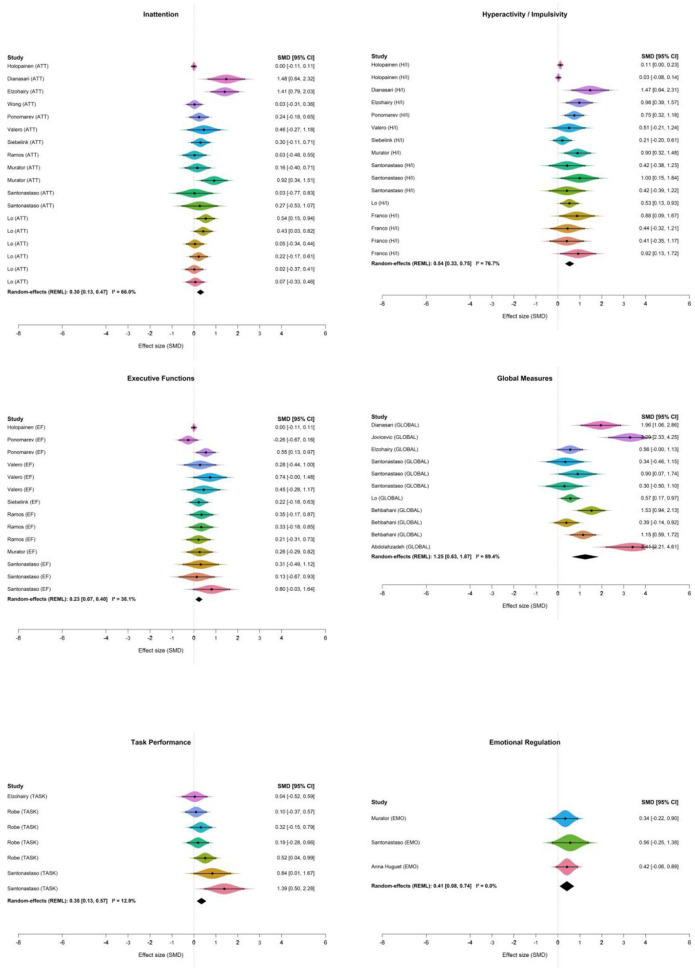
Forest plots by population subgroup.

Regarding consistency, the children subgroup exhibited moderate heterogeneity τ = 0.27 (I^2^ = 43%), whereas the adolescent subgroup showed substantial heterogeneity τ = 0.67 (I^2^ = 80%). These findings indicate greater variability in trial outcomes among adolescent samples. The forest plot of age subgroups is shown in Figure 6. The posterior density plots of the subgroups can be found in Supplementary file S3. The specific data of the subgroups can be found in Supplementary file S4.

#### 3.4.4 Moderation analysis by dosage

**Figure 7:**
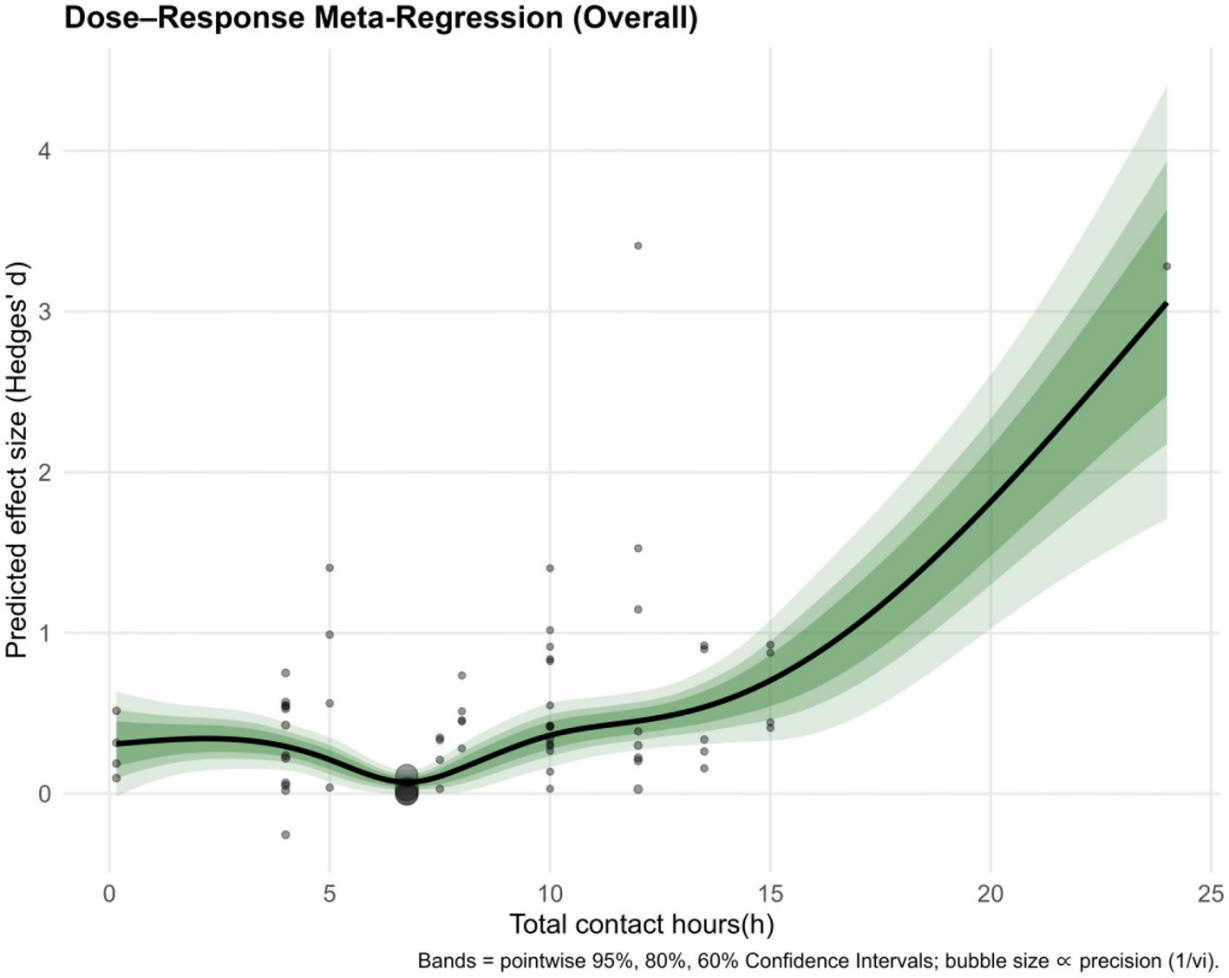
Moderation analysis by dosage (overall).

A generalized additive model (GAM) identified a non-linear association between total contact hours and Hedges' g (smooth term edf = 4.70, *F* = 7.62, *p* = 8.64 × 10^−6^), with adjusted R^2^ = 0.282 and deviance explained = 43.5% (*n* = 66; Figure 7). Predicted effects grew stronger as total contact hours increased across the observed range, though uncertainty in the estimates also rose toward the upper end due to sparser data. The shaded regions represent 95%, 80%, and 60% confidence intervals, which provide an indication of the uncertainty around the regression line at various levels. This dose–response relationship indicates that the intervention's effectiveness may improve with more extended contact time.

We explored how the number of total contact hours (“Sessions”) relates to outcomes across six domains using a generalized additive model (GAM). Notably, hyperactivity/impulsivity exhibited the most striking pattern, a U-shaped curve with clear improvement emerging after 7–8 h of contact. This effect was statistically robust (edf = 2.99, *F* = 6.14, *p* = 0.0086), accounting for a substantial 68.5% of variance. The intercept coefficient (0.437, *p* = 0.000232) further underscored the strength of the effect at higher doses. Global symptoms followed a more linear, upward trend, hinting at improvement with increased exposure. While not conventionally significant (*p* = 0.0652), the model explained a respectable 50.3% of variance, with an intercept of 1.0893 (*p* = 0.000888), suggesting potential benefits that merit further study.

Task performance also showed promising results, particularly after the 9–10 h mark, with the model explaining over 73% of the variation. Though the effect just missed conventional significance (*p* = 0.0773), the sharp upward trend and intercept (0.47266, *p* = 0.00697) indicate that extended exposure may enhance performance on targeted tasks. In contrast, inattention presented a more diffuse picture. The relationship between sessions and outcomes was weak and scattered (*p* = 0.273), explaining only 6% of the variance. The small but significant intercept (0.1785, *p* = 0.0193) showed little modulation with contact duration.

Executive functions followed a similarly ambiguous pattern—no significant trend emerged (*p* = 0.263), and variance explained was negligible, even slightly negative. The intercept (0.13343, *p* = 0.0642) reached marginal significance, but the overall pattern suggests limited impact from session length. Lastly, no credible association was observed between contact hours and emotion regulation outcomes. Estimates in this domain were relatively imprecise and highly variable, underscoring the need for additional data before firm conclusions can be drawn.

**Figure 8:**
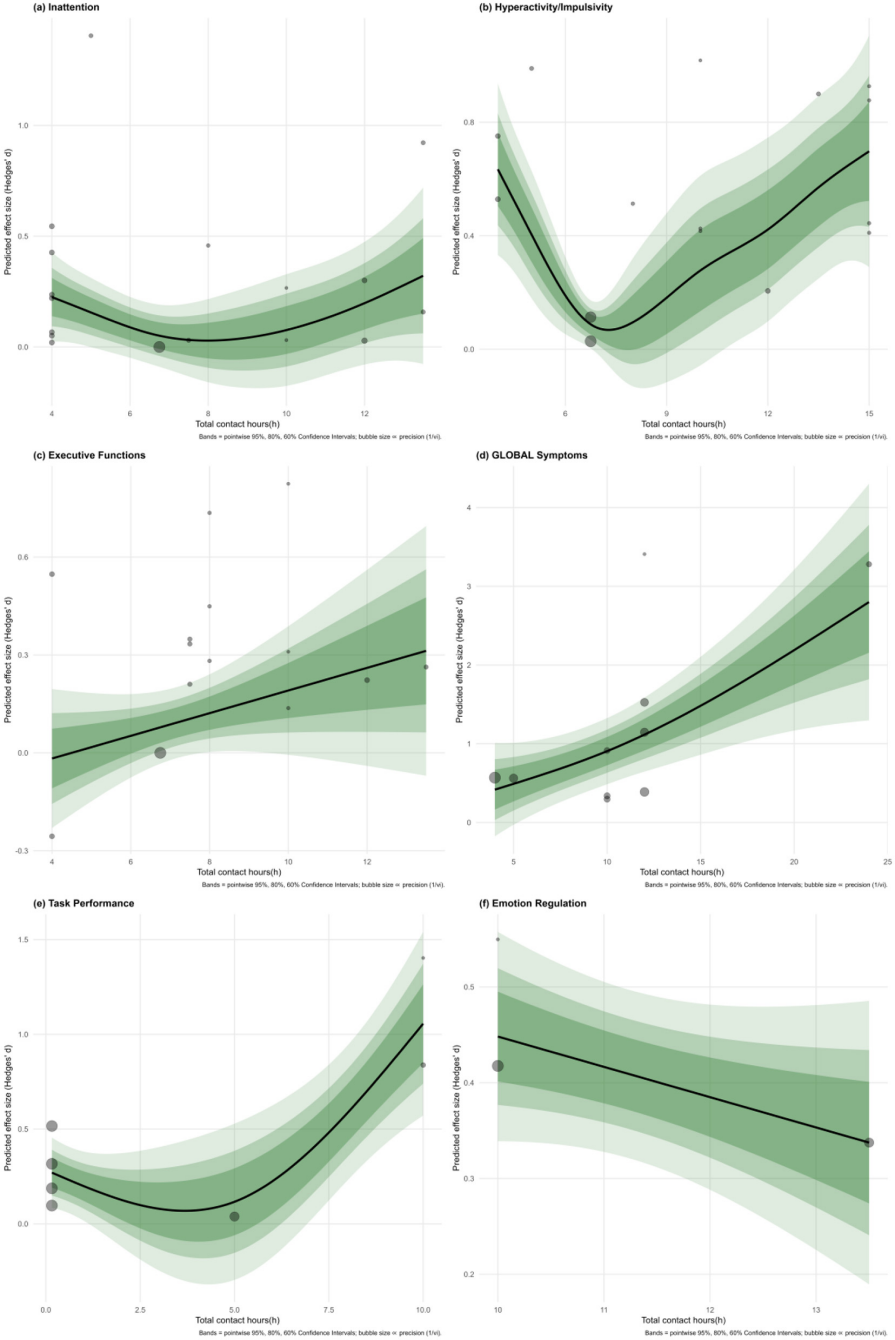
By domain moderation analysis.

In sum, while more sessions clearly support gains in hyperactivity/impulsivity, global symptoms, and task performance, their effect on attention, executive function, and emotional regulation remains ambiguous. The subgroup by domain dose analysis is shown in Figure 8, and the specific data are shown in Supplementary file S5.

#### 3.4.5 Sensitivity analysis

**Figure 9:**
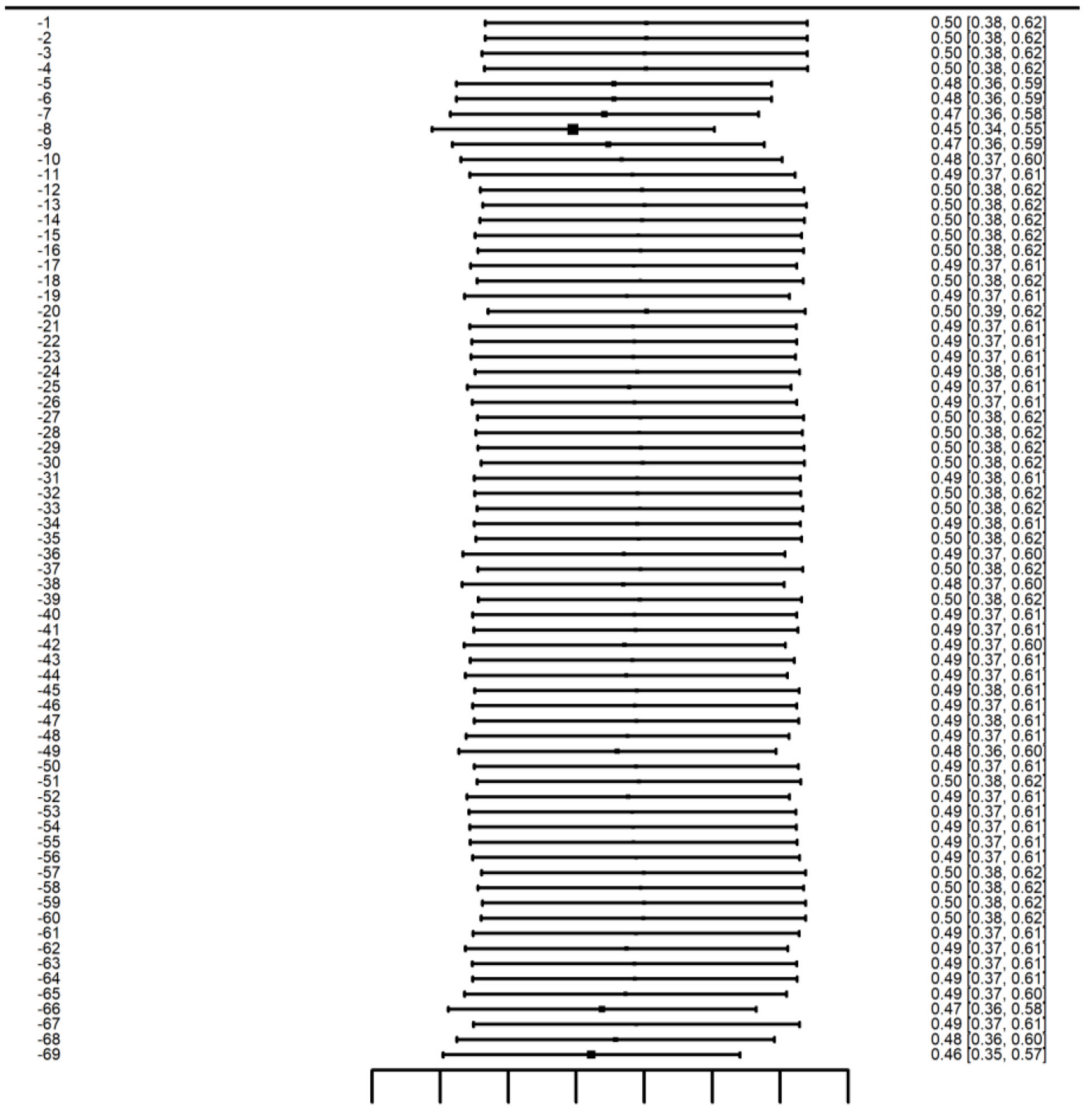
Leave-one-out sensitivity analysis.

In leave-one-out sensitivity analyses, the pooled effect size (Hedges' g) varied only slightly when each study was omitted in turn, ranging from approximately 0.45 to 0.50, indicating that the overall finding was robust. In all iterations, the effect remained positive and statistically significant (*p* < 0.001), confirming that the direction and significance of the effect were preserved regardless of which single study was removed. No individual study had a disproportionate influence on the summary effect, omission of any one study did not substantially alter the pooled estimate or its significance. The largest change in the pooled effect was observed upon excluding one study, which lowered the pooled Hedges' g from 0.50 to 0.45. However, even in this case the effect remained statistically significant and the direction of effect was unchanged. Sensitivity analysis is presented in Figure 9. The specific data of the sensitivity analysis can be found in Supplementary file S6.

#### 3.4.6 Publication bias

**Figure 10:**
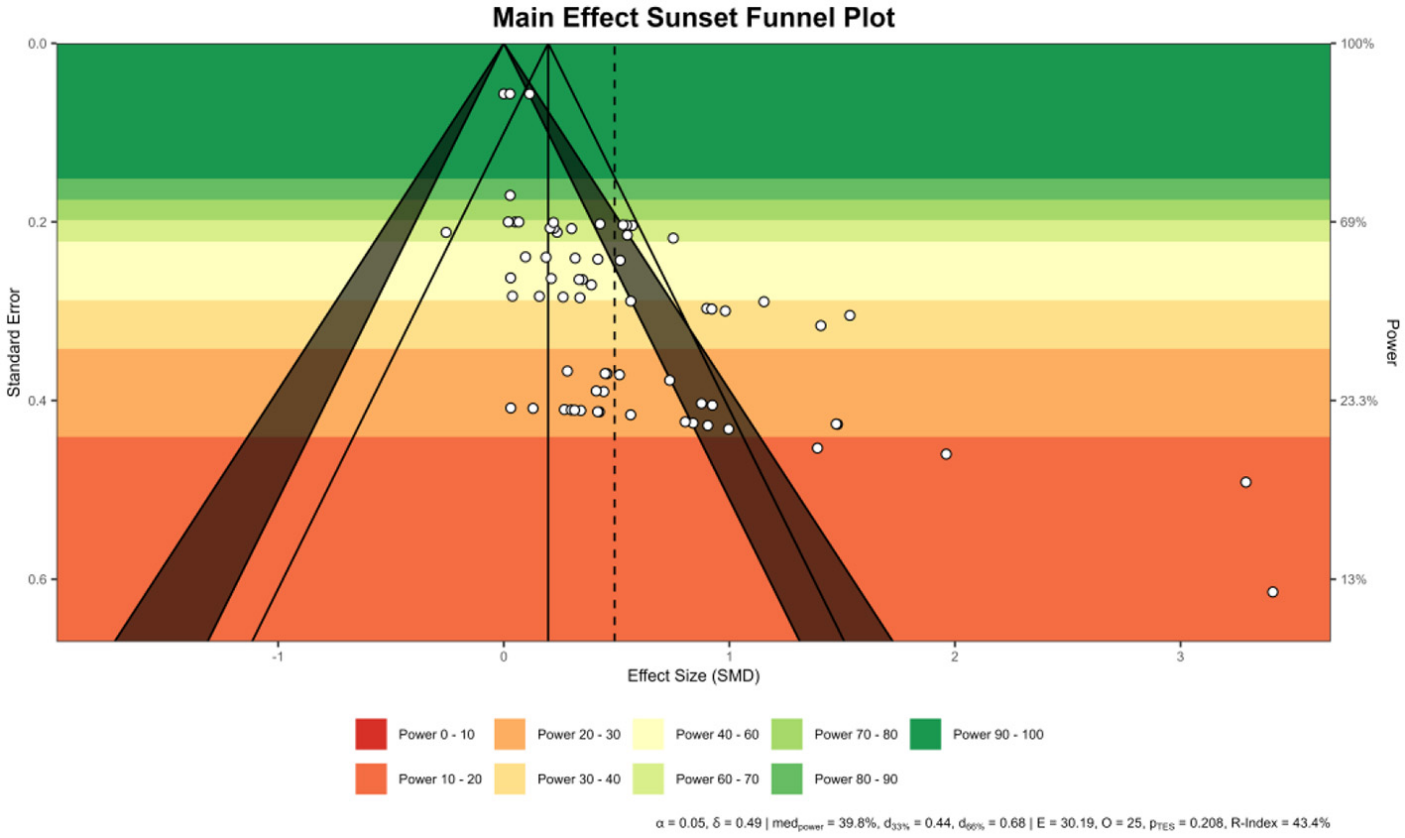
Sunset funnel and trim-and-fill (overall).

Egger's regression tests indicated evidence of small-study effects (potential publication bias) in most outcome domains except emotion regulation. Specifically, potential publication bias was observed for Inattention (z = 3.12, *p* = 0.0018), Hyperactivity/Impulsivity (z = 6.11, *p* < 0.0001), Executive Function (z = 2.82, *p* = 0.0048), Task Performance (z = 2.44, *p* = 0.0148), and Global functioning (z = 2.94, *p* = 0.0033). For Emotion Regulation, the results (z = 0.30, *p* = 0.7608) suggested an absence of publication bias. To further examine developmental patterns, Egger's tests within age strata were also significant. Children ≤ 10 years (z = 2.36, *p* = 0.023) and adolescents >10 years (z = 5.88, *p* < 0.00001), indicating asymmetry in both strata, more pronounced among adolescents. To further examine the robustness of the findings, we performed trim-and-fill analyses and visualized the results using sunset funnel plots (Figure 10). Sunset funnel plots illustrate the distribution of studies by effect size and standard error, with colored bands indicating statistical power levels (red = low power, green = high power). Although Egger's tests revealed funnel-plot asymmetry, the trim-and-fill procedure imputed only a limited number of potentially missing studies, and the adjusted pooled effect sizes were only slightly attenuated. Importantly, the overall effects remained statistically significant across domains, indicating that potential publication bias had minimal influence on the robustness of the meta-analytic results. The funnel plots of each subgroup (including age strata) are shown in Supplementary file S7.

## 4 Discussion

This is the first Bayesian meta-analysis examining the effects of Mindfulness-Based Interventions (MBIs) on symptoms in children and adolescents with ADHD. This systematic review and meta-analysis consolidate evidence regarding (1) The impact of MBIs on ADHD symptoms in children and adolescents and (2) the influence of MBI dosage on symptomatic outcomes in children and adolescents.

### 4.1 Summary of findings

This Bayesian meta-analysis indicates that mindfulness-based interventions (MBIs) yield moderate improvements in ADHD symptoms in children and adolescents. Subgroup analyses show significant improvements in hyperactivity/impulsivity and global ADHD ratings, followed by attention, executive function, and task performance, though the effect on emotion regulation is not significant. Age-stratified analyses show larger effects in adolescents than in children. The dose-response analysis reveals that longer intervention durations are associated with greater symptom improvements, with optimal effects observed when intervention time exceeds 10 h. Overall, MBIs represent an effective complementary treatment for ADHD, particularly for families seeking non-pharmacological options.

### 4.2 The effects of MBIs on ADHD

The effects of MBIs on ADHD symptoms were domain-specific, with the most substantial improvements observed in hyperactivity/impulsivity and global symptoms, modest benefits in attention, executive functions, and task performance, while effects on emotion regulation were inconclusive given imprecision and a 95% credible interval crossing the null. However, considerable heterogeneity was present, stemming not only from differences among symptom domains but also from variability in the populations. Nevertheless, the sensitivity analysis validates the robustness of our results. These findings align with previous studies conducted in pediatric populations, which also reported stronger effects on behavioral symptoms than on emotional domains (van de Weijer-Bergsma et al., 2012; van der Oord et al., 2012). Several interrelated mechanisms likely underlie these improvements. Mindfulness practice fundamentally trains attention regulation and inhibitory control, skills that are directly relevant to ADHD symptomatology. At a psychological level, mindfulness teaches individuals to observe thoughts and impulses non-reactively, which helps “override” automatic impulsive responses and replace them with more deliberate choices (Schuman-Olivier et al., 2020). At the neurobiological level, mindfulness appears to strengthen brain networks responsible for executive attention and cognitive control. Brain imaging research shows that after mindfulness training, individuals with ADHD exhibit increased activation in key attention-regulation regions, such as the frontoparietal network (including the inferior parietal lobules and precuneus) and the insular cortex, during cognitive tasks (Bachmann et al., 2018; Charness et al., 2024; Gu et al., 2021). In parallel, mindfulness training may down-regulate the brain's default mode network (DMN), which is associated with mind wandering and distractibility, thus reducing internal distractions (Hölzel et al., 2011; Xu et al., 2022). Moreover, mindfulness induces neuroplastic changes in brain structures linked to attention and self-regulation. In experienced practitioners, researchers have found structural and functional alterations in several key brain regions. These include the anterior cingulate cortex, a region important for attentional control, alongside the prefrontal cortex and fronto-striatal circuits, which are critical for executive function and inhibitory control (Garland et al., 2014; Hölzel et al., 2011; Xu et al., 2025). Overall, MBIs yield the greatest gains in hyperactivity/impulsivity and global symptoms, smaller improvements in attention, executive functions and task performance, and no credible improvements in emotion regulation.

The absence of a credible effect on emotion regulation warrants specific consideration. In youth ADHD, syntheses of randomized trials suggest that MBIs can reduce core ADHD symptoms, whereas downstream effects on broader emotional/behavioral problems are comparatively smaller or less consistent (Lee et al., 2022). One plausible explanation is domain specificity: standard mindfulness training primarily strengthens attentional monitoring and top-down control capacities that align more closely with “cool” executive functions than with affectively laden “hot” executive processes involved in emotional responding (Zelazo and Carlson, 2012). In parallel, emotional dysregulation in ADHD is multifaceted and clinically consequential, with partially distinct neurobehavioral pathways, which may make it less responsive to non-specific skills training (Shaw et al., 2014). Accordingly, measurable change in emotion regulation may require either a higher intervention “dose” or explicit affect-focused ingredients that more directly engage emotion-regulatory mechanisms.

In our Age-subgroup analyses, MBIs benefited both children and adolescents with ADHD, with a consistent tendency toward stronger effects in adolescents than in younger children. Converging evidence from the youth MBI literature supports this developmental gradient. In contrast, interventions focused solely on children, while still beneficial, tend to yield more modest effects and appear to depend more heavily on the simultaneous use of mindful-parenting strategies (van de Weijer-Bergsma et al., 2012; van der Oord et al., 2012). In school-based syntheses across youth, late-adolescent samples have yielded larger pre–post effects than middle-childhood samples, suggesting age can moderate responsiveness to mindfulness curricula, a trend that aligns with our subgroup pattern (McKeering and Hwang, 2019). Meta-analytic work dedicated to preadolescents likewise indicates that, while MBIs confer small but reliable benefits on attention and self-regulation, pooled effects are generally modest in this younger band again consistent with our observation that gains strengthen in older youth (Kander et al., 2024). Early meta-analytic overviews of youth MBIs also noted that average sample age showed a positive (albeit non-significant) association with effects, pointing in the same direction (Zoogman et al., 2015). A plausible explanation is developmental, mindfulness skills such as meta-awareness, sustained attention, and inhibitory control recruit prefrontal and frontoparietal networks (Tang et al., 2015) that undergo protracted maturation across adolescence (Fair et al., 2007; Luna et al., 2015). As executive control systems specialize and strengthen, adolescents may be better positioned to translate mindfulness practice into measurable behavioral change. However, because the same age cut-off was applied uniformly across heterogeneous outcome domains and stratification reduced the amount of information within each stratum (with several domain-by-age cells containing too few trials/effect sizes for stable estimation), any apparent cross-domain subgroup pattern should be interpreted cautiously and regarded as exploratory (hypothesis-generating) rather than confirmatory.

In short, MBIs address ADHD on multiple levels. They retrain attentional habits, cultivate more deliberate control over impulses, and engage prefrontal–frontoparietal circuits that underpin sustained attention and executive functioning (Tang et al., 2015). With regular practice, individuals with ADHD can develop greater mental discipline and cognitive stability, helping to explain improvements across both behavioral symptoms and cognitive domains. These findings suggest that MBI programs achieve the greatest gains when they are tailored to developmental stage and delivered in an age-appropriate manner. MBIs should also be symptom-targeted for children and adolescents with ADHD. Notably, evidence is not uniform across settings. In a large school-based cluster randomized trial delivering a universal 9-week mindfulness curriculum to whole classrooms, no reduction in elevated ADHD symptoms was observed relative to active or passive controls, suggesting that brief, class-wide programs may be insufficient to shift ADHD-related outcomes at scale (Holopainen et al., 2025). This contrasts with our adolescent subgroup trend and supports the view that dose and delivery format are critical moderators. Similar boundary conditions emerge when the comparison is a strong active control. In MindChamp, family MBI added to care-as-usual did not outperform care-as-usual at the group level, and in a head-to-head trial MBI and CBT produced comparable improvements without between-group differences (Siebelink et al., 2022; Wong et al., 2023). Together, these findings indicate that MBIs may yield context-dependent benefits, larger under adequate contact time and targeted clinical delivery, but attenuated in universal formats or against potent active controls.

We found considerable between-study heterogeneity (τ ≈ 0.40; I^2^ ≈ 82%) in effect sizes. They included trials varied in participants, intervention formats, and control comparisons. For instance, effects were generally larger in studies comparing MBIs to waitlist controls and smaller in those with active treatment controls. Differences in outcome measures also likely contributed to variability. Psychosocial intervention studies often find smaller gains on blinded or objective measures than on unblinded parent ratings (Daley et al., 2014; Sonuga-Barke et al., 2013). Notably, heterogeneity was reduced in subgroup analyses by symptom domain (e.g., I^2^ = 52.7% for attention, 50.0% for hyperactivity/impulsivity), indicating that differential effects across domains contributed significantly to the overall variation. Encouragingly, our leave-one-out analysis showed that the pooled effect remained around ~0.5 even when each study was omitted in turn, indicating that the overall benefit is not driven by any single trial. The domain-specific impacts of MBIs underscore the importance of developing more targeted and developmentally appropriate interventions.

There was also evidence of small-study publication bias. Egger's test was significant, indicating smaller studies with larger effects might be over-represented. We assessed potential publication bias using the trim-and-fill method, which accounts for hypothetical missing studies. The adjusted effect remained positive and was only slightly attenuated compared to the observed estimate. Importantly, our Bayesian model's use of prior information likely mitigated the influence of outliers, yielding a more conservative pooled estimate. Thus, while the exact magnitude of benefit should be interpreted with some caution, the convergence of evidence indicates that MBIs do confer a real positive effect for ADHD symptoms.

Future research should prioritize four areas. First, tailor mindfulness interventions to developmental and symptom profiles, using techniques like emotion labeling for dysregulation. Second, establish optimal dosage through varying parameters like duration and frequency, and test boosters to sustain effects. Third, enhance methodological rigor with active controls, preregistration, blinding, and multi-method assessment. Large multi-site trials are needed to examine moderators and improve generalizability. Finally, future studies should embrace open science frameworks and advanced analytical techniques, such as Bayesian statistics, to strengthen the validity and cumulative value of empirical findings.

### 4.3 Dose–response relationship

A notable secondary finding was a non-linear dose–response relationship between intervention intensity and outcomes. Meta-regression indicated that longer total exposure to mindfulness (especially beyond the typical ~8-week/~10-h program duration) was associated with larger symptom improvements. In practical terms, very brief or low-intensity programs yielded minimal benefits (Taylor et al., 2021), whereas more intensive courses (approaching or exceeding standard mindfulness training doses) led to much greater gains. Within the range of doses in our data (up to ~20–25 h of instructor-led training), no clear plateau was observed, as the effect size continued to increase with additional hours. These observations are consistent with broader MBIs literature showing dose-related benefits for mindfulness skills and selected outcomes. A meta-analysis of MBSR/MBCT found a small-to-moderate, significant association between amount of home practice and post-treatment outcomes across 28 studies, indicating that “doing more” confers additional gains beyond simply enrolling in a course (Parsons et al., 2017). Meanwhile, evaluations of classic programs found a correlation between more minutes of home practice during 8-week courses and greater improvements in mindfulness, symptom reduction, and wellbeing, thereby providing evidence for a dose-response interpretation linked to actual engagement rather than nominal enrollment (Carmody and Baer, 2008). Additionally, follow-up data suggest that without continued practice or booster sessions, initial gains may wane within a few months post-intervention (Lee et al., 2022; van de Weijer-Bergsma et al., 2012). Since most of the included trials assessed outcomes only at post-treatment and provided limited long-term data, our findings indicate that greater intervention dose is associated with larger endpoint improvements, whereas previous research cautions that such benefits are not self-sustaining. Taken together, these strands suggest a pragmatic model, sufficient initial intensity is needed to achieve clinically meaningful change, while continued practice or booster sessions are likely required to maintain these improvements beyond the immediate intervention period. Overall, our results highlight that “more is more” up to practical limits, suggesting that adequate MBI dosage is critical for achieving meaningful improvements in ADHD symptoms.

### 4.4 Strengths and limitations

This study provides the first Bayesian meta-analysis dedicated to mindfulness-based interventions (MBIs) for children and adolescents with ADHD. A Bayesian random-effects framework with weakly informative priors was used, and MCMC convergence was explicitly monitored, strengthening inferential reliability. Clinically, the analysis separates effects by symptom domains (inattention; hyperactivity/impulsivity; executive functions; global ratings; task performance; emotion regulation), which helps identify where MBIs are most likely to yield benefits. A further contribution is the non-linear dose–response modeling (GAM), which uncovers practically useful exposure patterns and thresholds for contact hours that would be difficult to detect with simpler approaches. Together, these features translate statistical rigor into actionable guidance for program design and clinical decision-making in pediatric ADHD. Moreover, we conducted age-stratified analyses to add a developmental lens to the evidence. This approach tests whether treatment gains vary by maturational stage while holding intervention characteristics and outcome definitions constant, thereby improving interpretability. Taken together with the domain-specific and dose response findings, this enhances external validity and provides clearer direction for age-appropriate program design and evaluation in pediatric ADHD.

Several factors may limit the certainty and generalizability of the pooled estimates. First, restricting inclusion to English-language publications may have introduced language bias and reduced representation of evidence from non-English-speaking regions. Second, most studies assessed outcomes immediately post-intervention; the scarcity of long-term follow-up data prevents firm conclusions about the durability of MBI-related benefits in the absence of continued practice. Third, although both individually randomized and cluster-randomized trials were included to broaden scope, the exclusion of non-randomized studies and qualitative research means that evidence from real-world, non-experimental contexts is not captured. Finally, and most importantly, confidence in the findings is tempered by the high risk of bias in many trials, particularly the lack of blinded outcome assessment (often involving subjective ratings). Together with evidence suggestive of small-study effects, which may reflect publication bias and/or other biases, the true effects may be smaller than the pooled estimates. Future adequately powered, methodologically rigorous RCTs incorporating blinded assessments and longer follow-up are needed to confirm these findings.

## 5 Conclusion

This Bayesian meta-analysis indicates that MBIs provide clinically meaningful benefits for children and adolescents with ADHD. The overall effect size (μ≈ 0.49, 95% CrI 0.37 to 0.62) is moderate, with notable improvements in hyperactivity/impulsivity and global symptom ratings, and more modest gains in inattention, executive functions, and task performance, while data regarding emotion regulation did not demonstrate a credible treatment benefit. Age-subgroup analyses suggest that adolescents tend to benefit more than children. A non-linear dose–response indicates that sufficiently intensive interventions yield greater benefits, highlighting the importance of adequate exposure. Despite considerable heterogeneity across trials, the positive effect of MBIs persisted across multiple analyses, supporting its credibility. Since a large proportion of the included trials had a high risk of bias, especially due to the lack of blinding in outcome assessment, our pooled estimates may overestimate the true benefits (especially for subjective symptom scores). This heterogeneity likely reflects differences in intervention protocols, participant characteristics, and outcome assessment methods. Taken together, MBIs constitute a useful psychosocial intervention for pediatric ADHD, especially for improving behavioral regulation, and a promising adjunct to standard treatments. Future work should delineate the populations with the greatest expected benefit and optimize delivery to enhance both effect size and durability.

## Data Availability

The original contributions presented in the study are included in the article/Supplementary material, further inquiries can be directed to the corresponding author.
