# Supplementary material for: Mindfulness-based interventions for children and adolescents with attention-deficit/hyperactivity disorder: a Bayesian meta-analysis of randomized controlled trials
Source: Front Psychol. 2026 Mar 11;17:1711994. doi: 10.3389/fpsyg.2026.1711994 (PMC13013061; doi:10.3389/fpsyg.2026.1711994)
Supplement: Supplementary file 1 [file Data_Sheet_1.ZIP › supplementary file/Supplementary file S3_Posterior_Densities_Subgroups.docx]

**Supplementary file S3:** Posterior Densities Subgroups


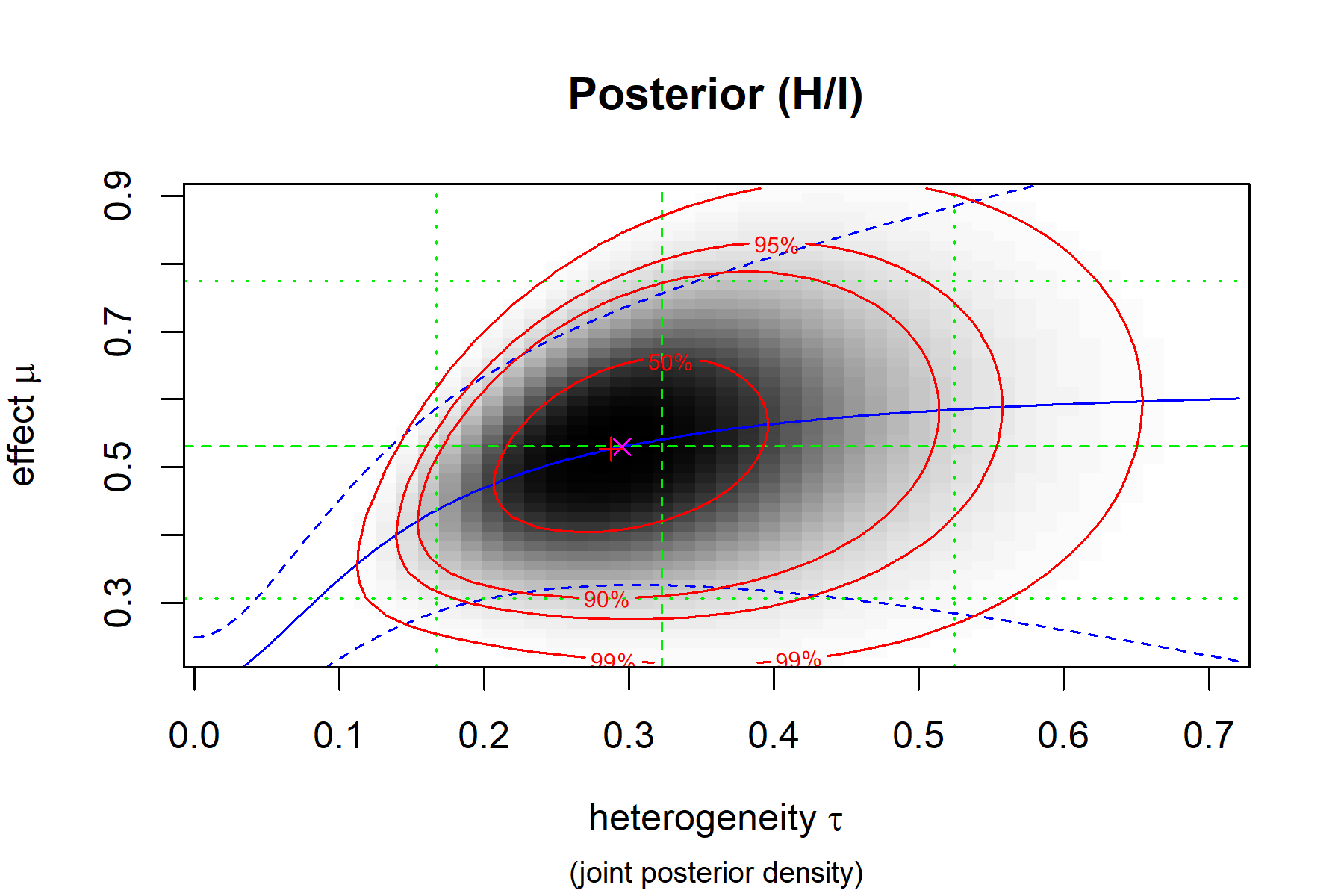


**Figure S1**. Posterior Hyperactivity/Impulsivity (H/I) joint μ–τ


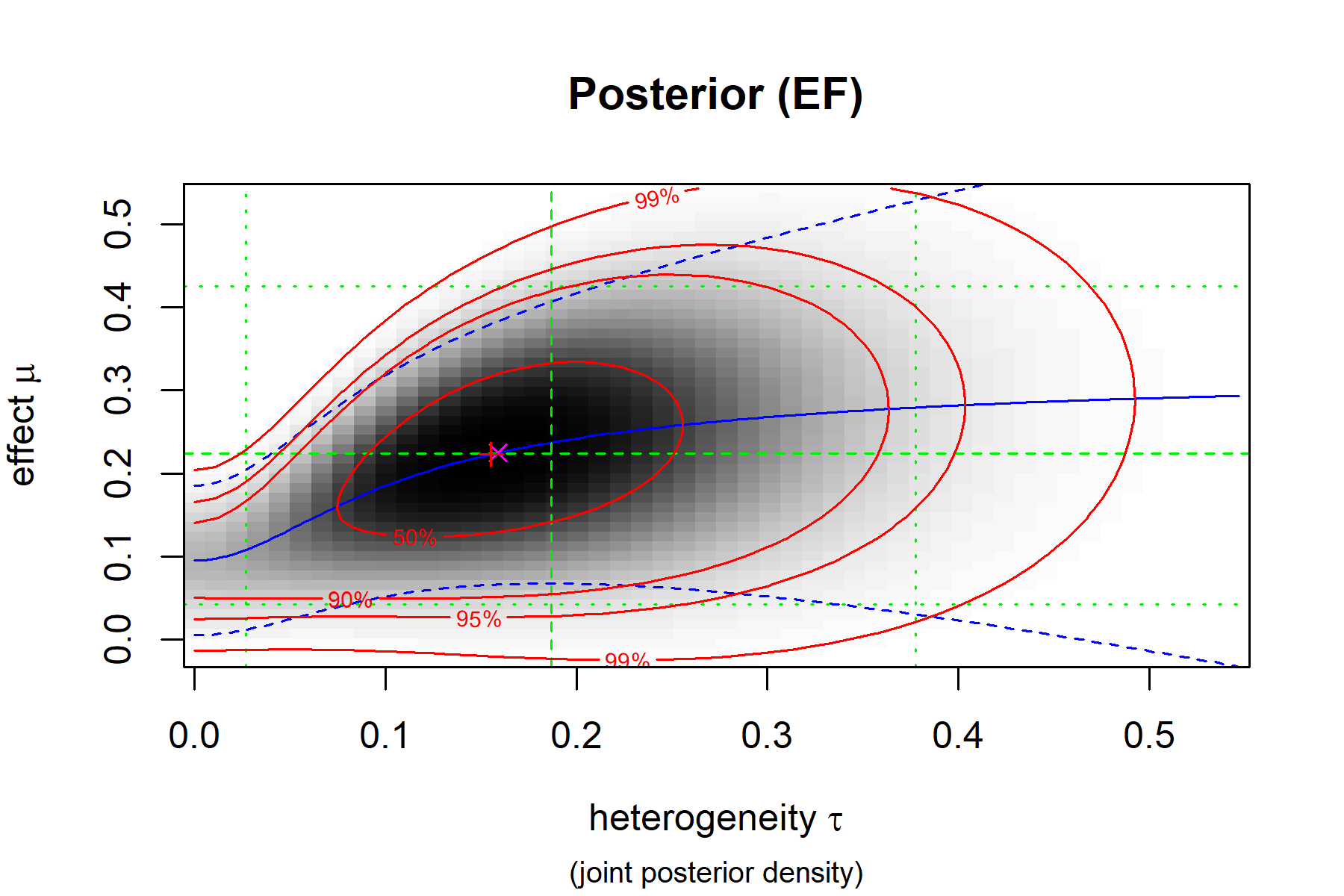


**Figure S2.** Posterior Executive Functions (EF) joint μ–τ


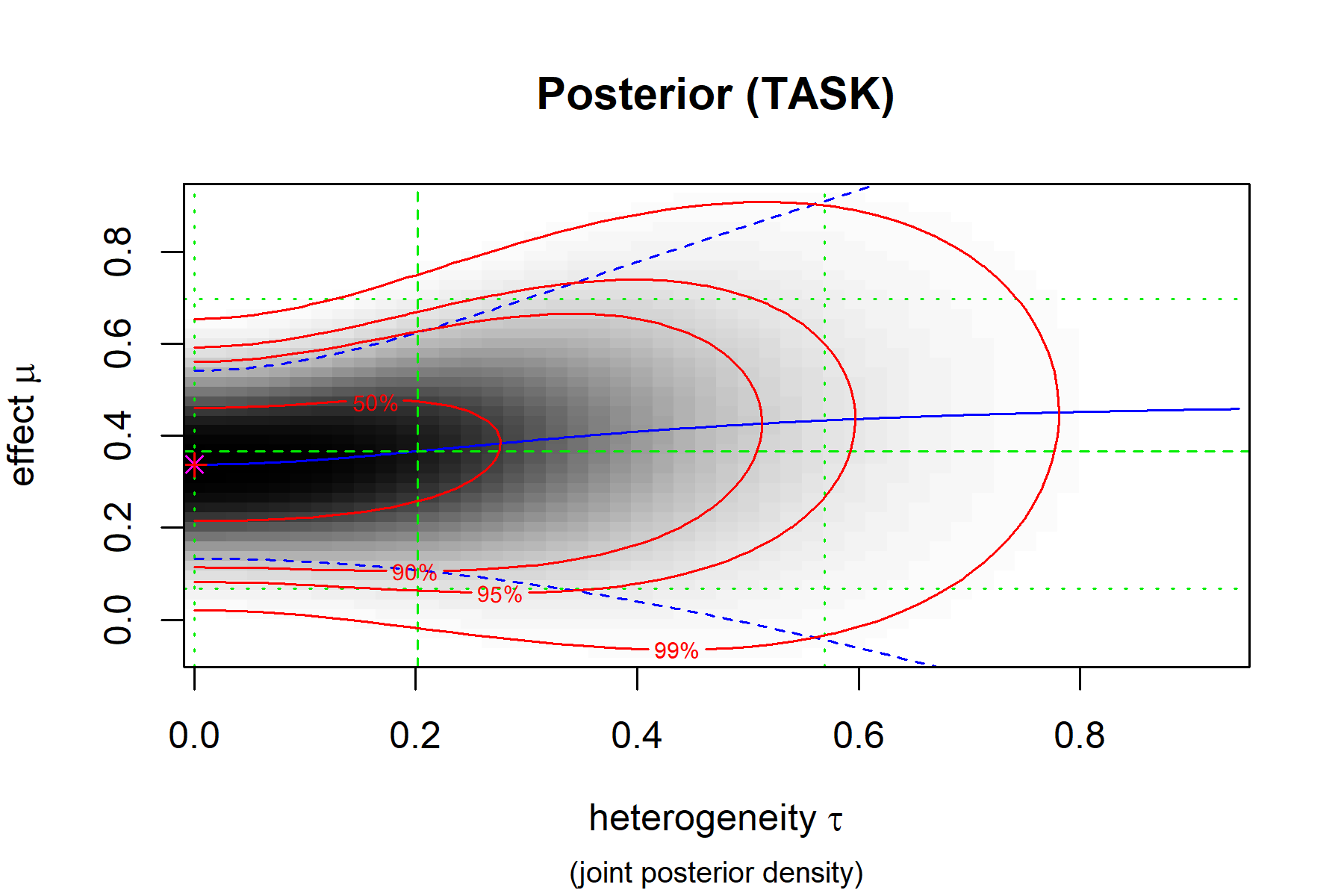


**Figure S3.** Posterior Task performance (TASK) joint μ–τ


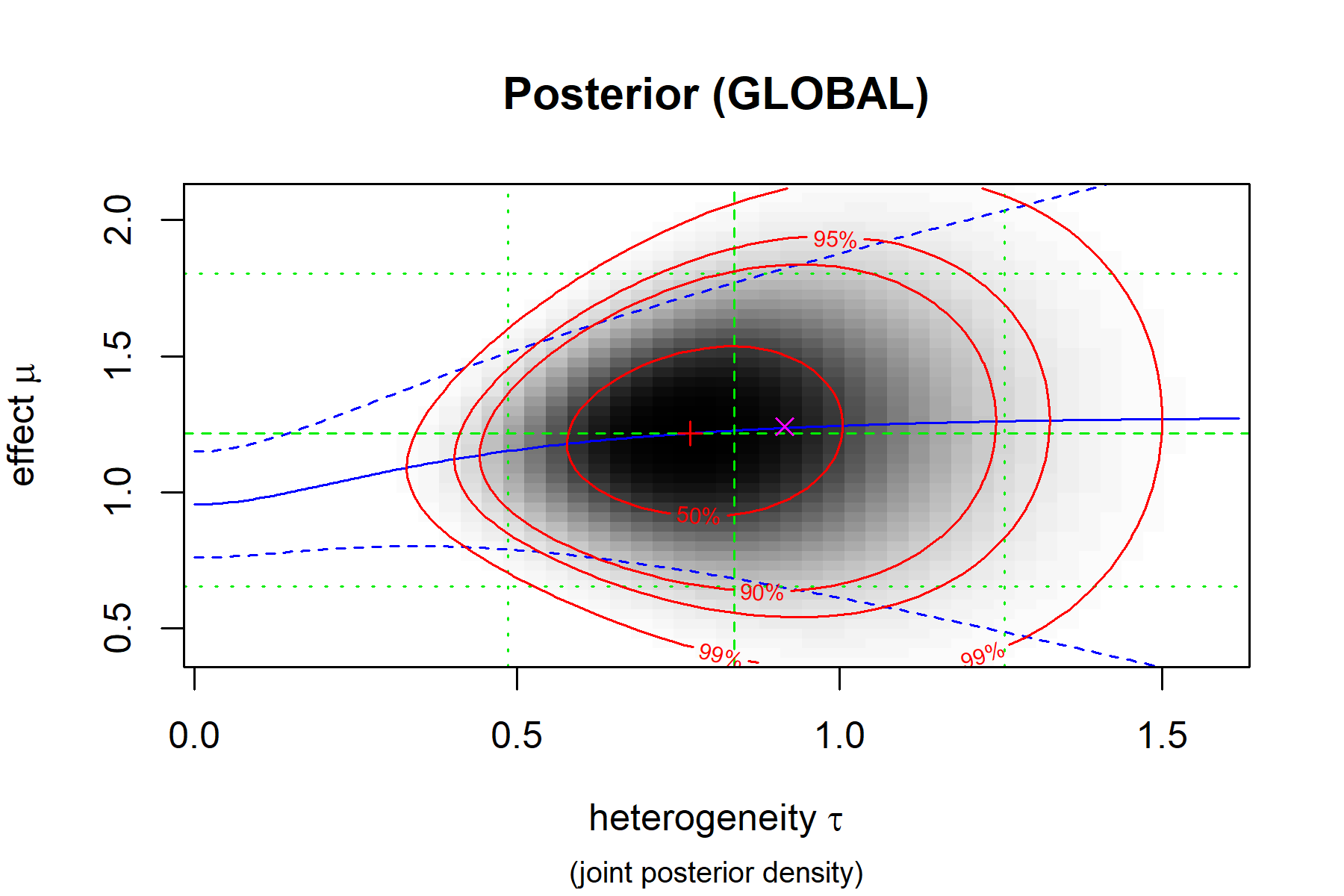


**Figure S4.** Posterior Global Measures (GLOBAL) joint μ–τ


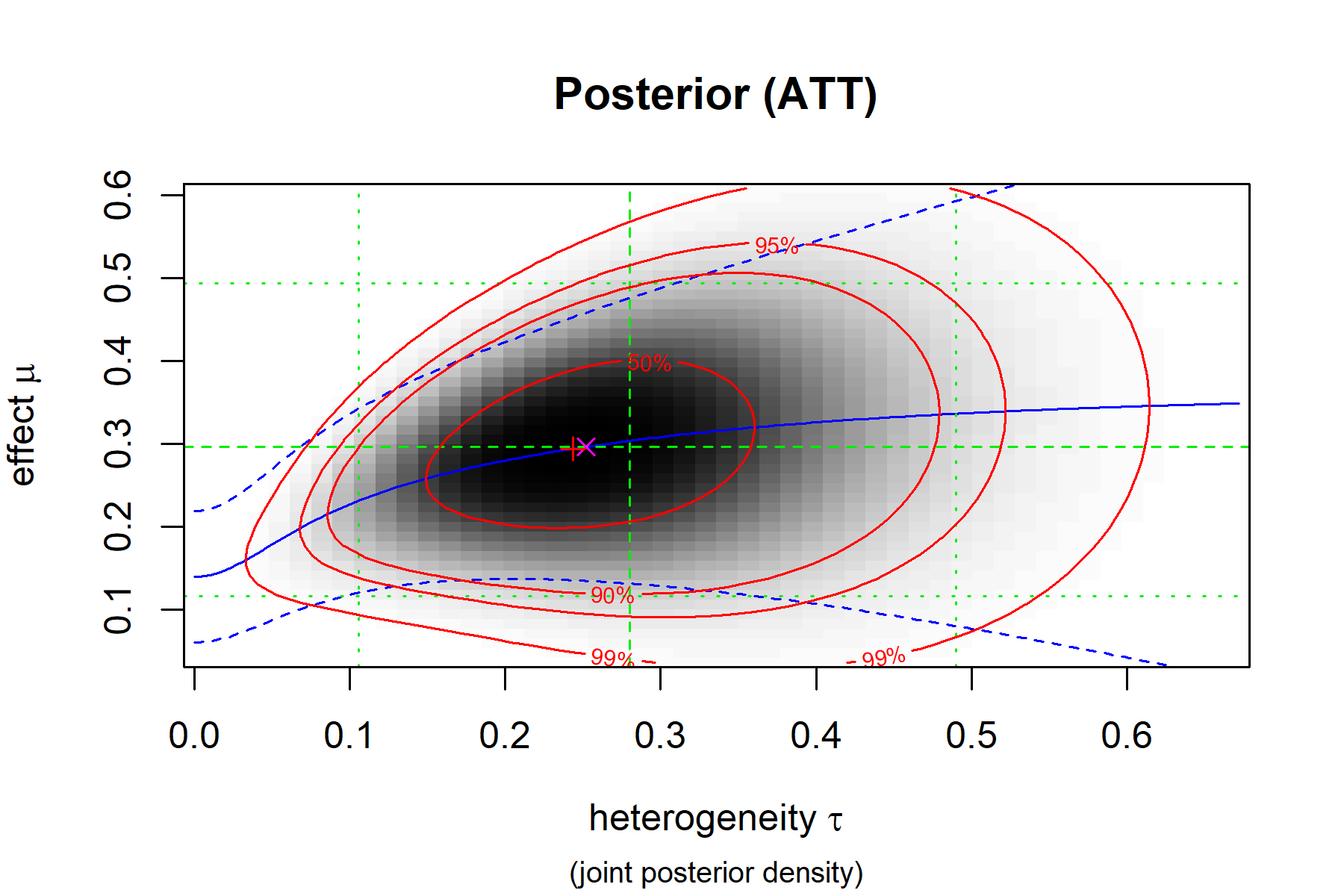


**Figure S5.** Posterior Inattention (ATT) joint μ–τ


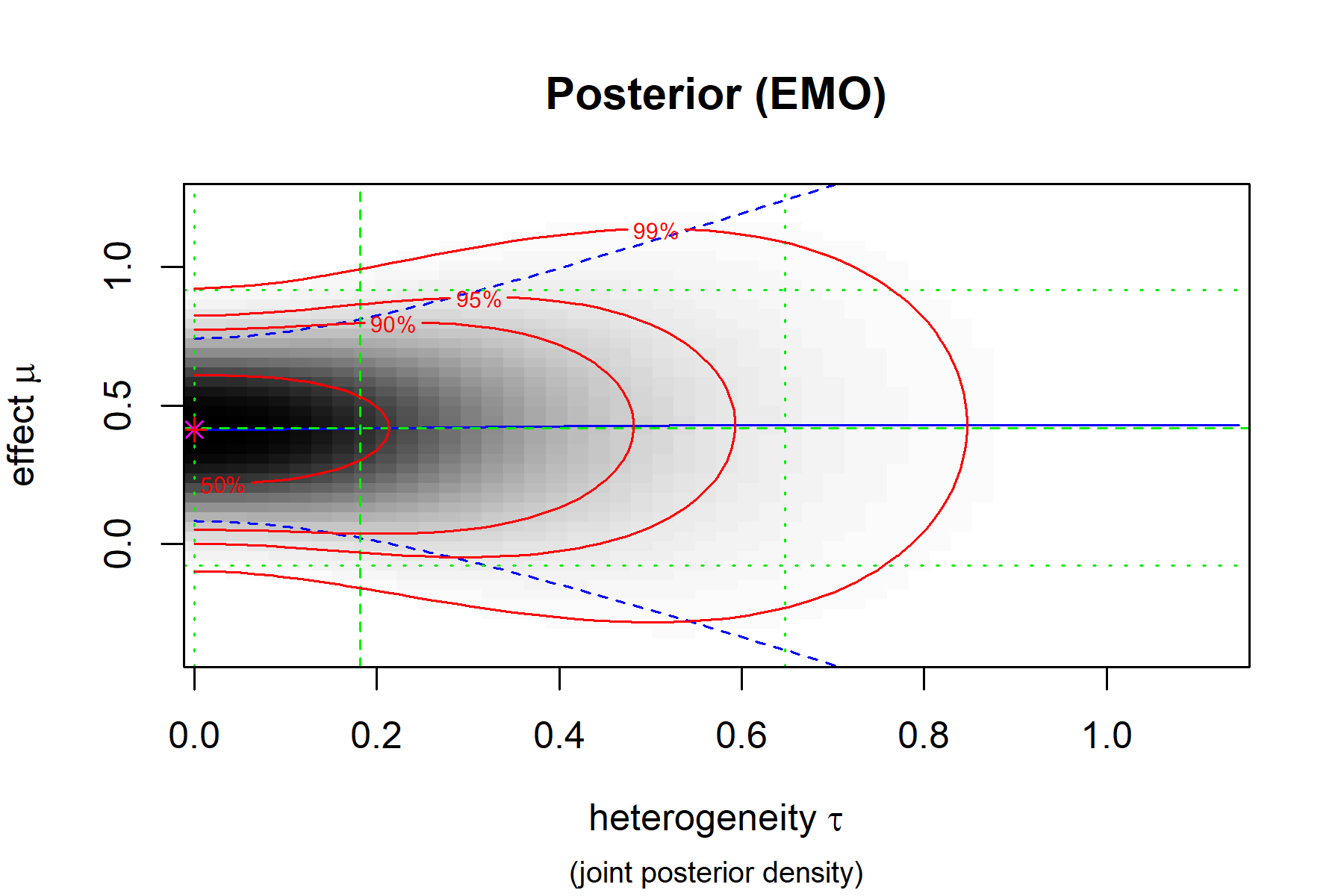


**Figure S6.** Posterior Emotion Regulation (EMO) joint μ–τ


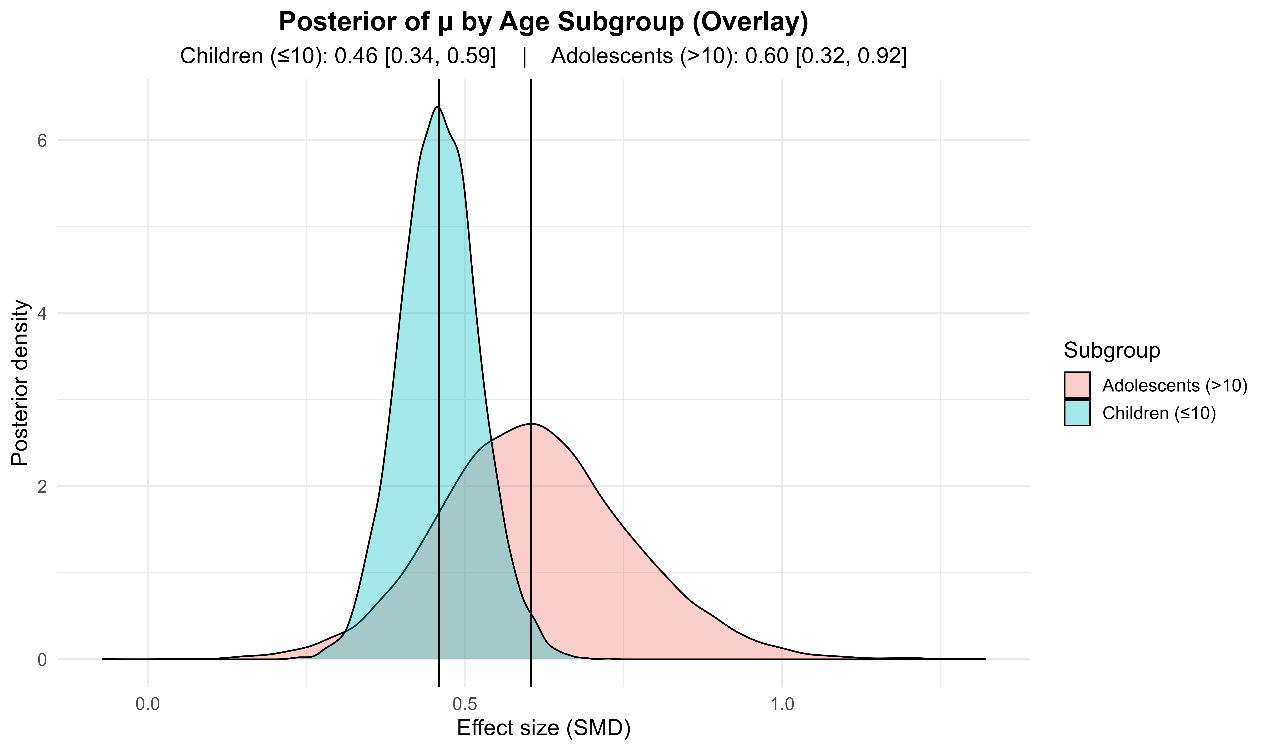


**Figure S7.** Posterior Population subgroup joint μ–τ
