# Supplementary material for: Mindfulness-based interventions for children and adolescents with attention-deficit/hyperactivity disorder: a Bayesian meta-analysis of randomized controlled trials
Source: Front Psychol. 2026 Mar 11;17:1711994. doi: 10.3389/fpsyg.2026.1711994 (PMC13013061; doi:10.3389/fpsyg.2026.1711994)
Supplement: Supplementary file 1 [file Data_Sheet_1.ZIP › supplementary file/Supplementary file S7_Sunset_Funnels_Subgroup.docx]

**Supplementary file S7**: Surgroup Sunset Funnels


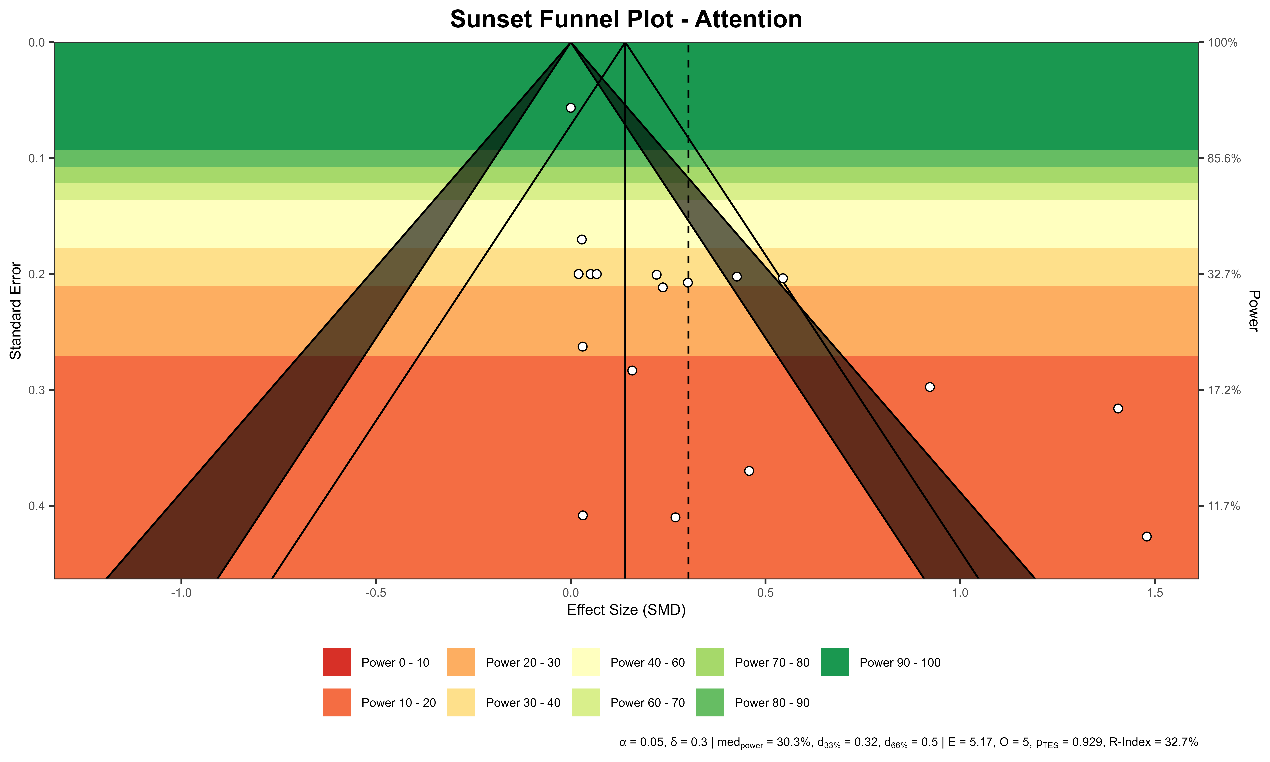


**FigureS1.** Forest plot for Attention (ATT)


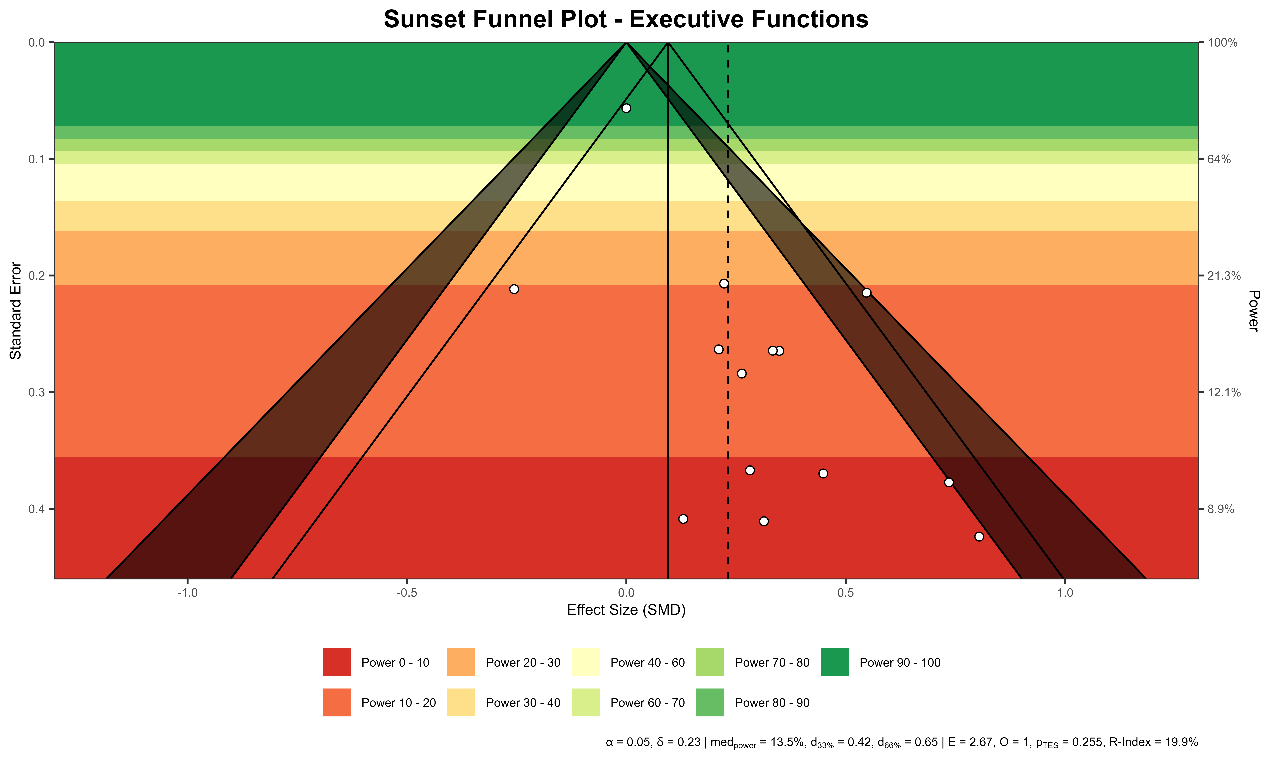


**FigureS2.** Forest plot for Executive Functions (EF)


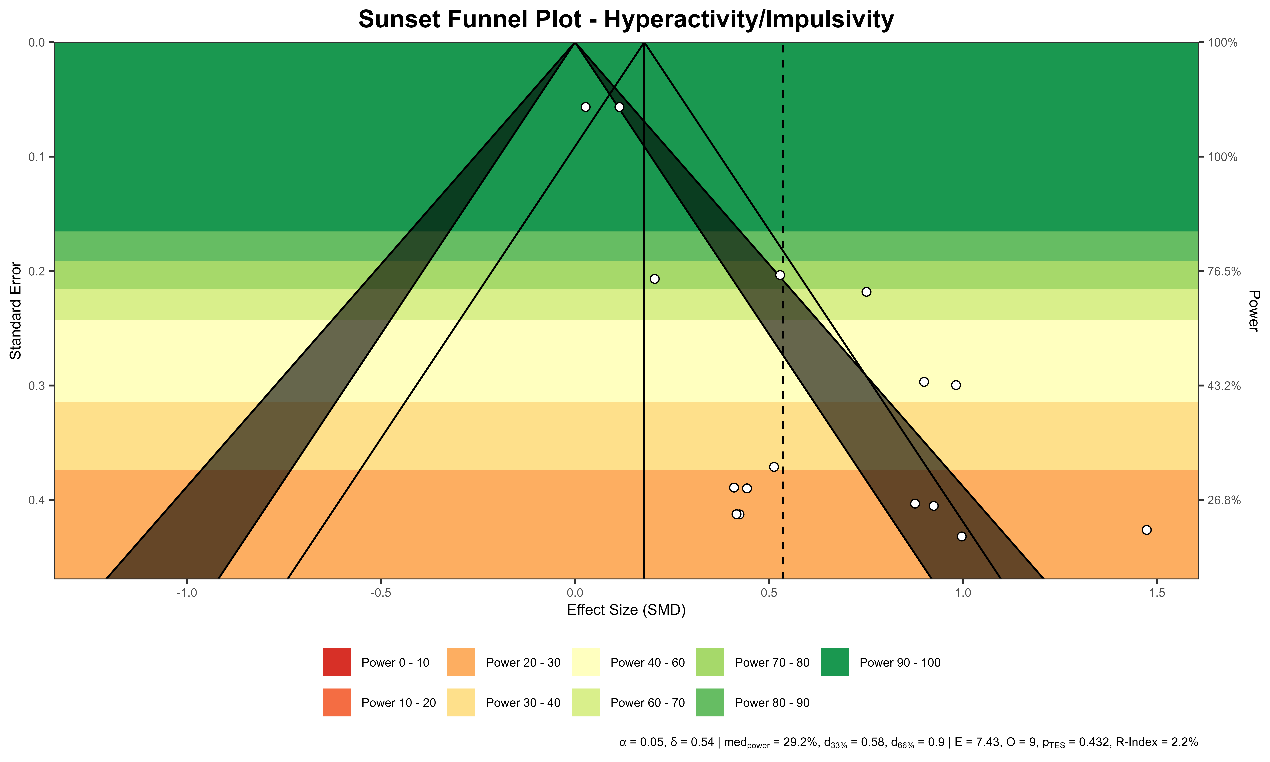


**FigureS3.** Forest plot for Hyperactivity/Impulsivity(H/I)


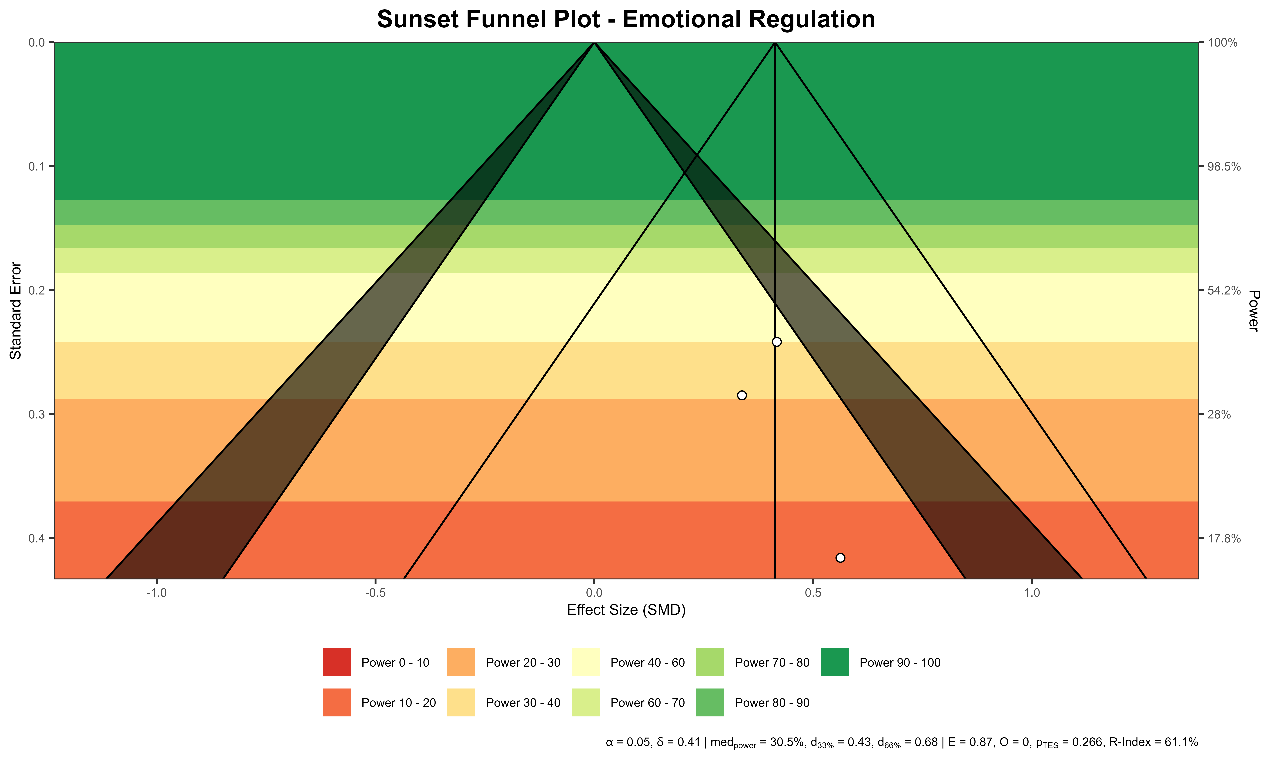


**FigureS4.** Forest plot for Emotion Regulation (EMO)


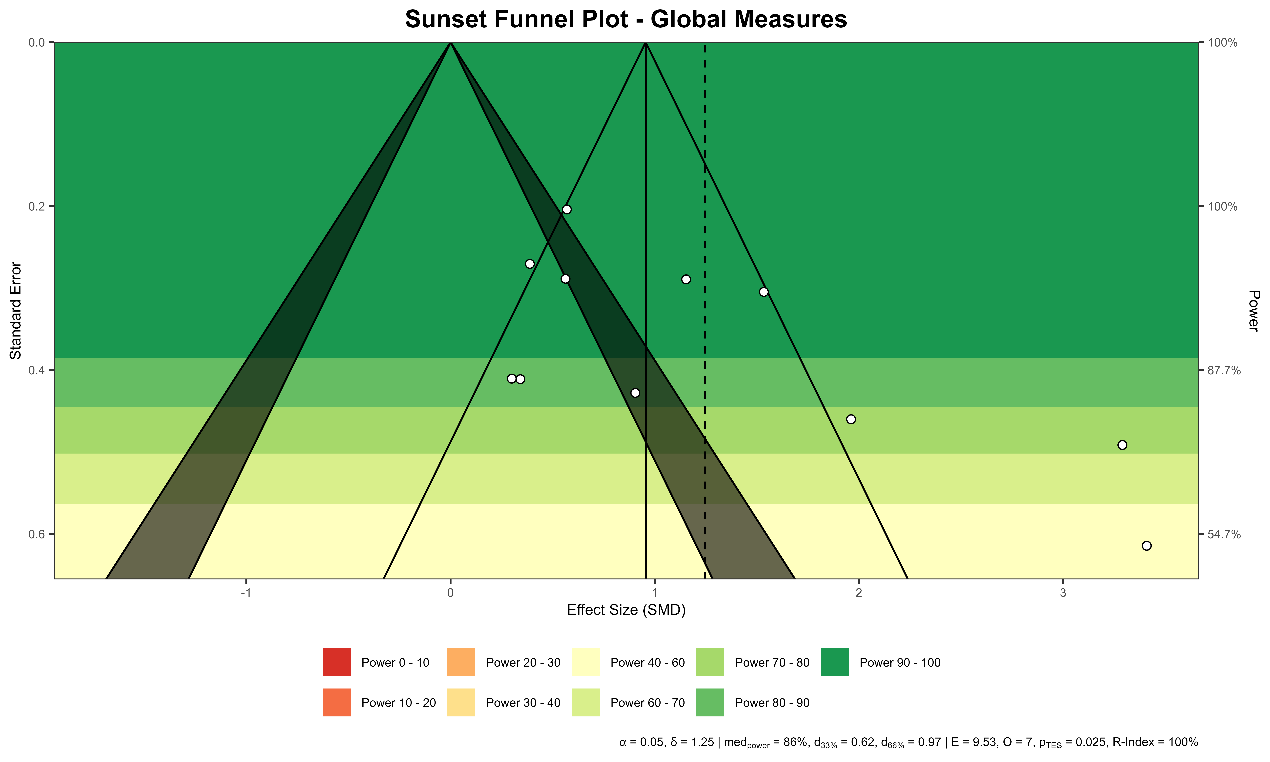


**FigureS5.** Forest plot for Global Measures (GLOBAL)


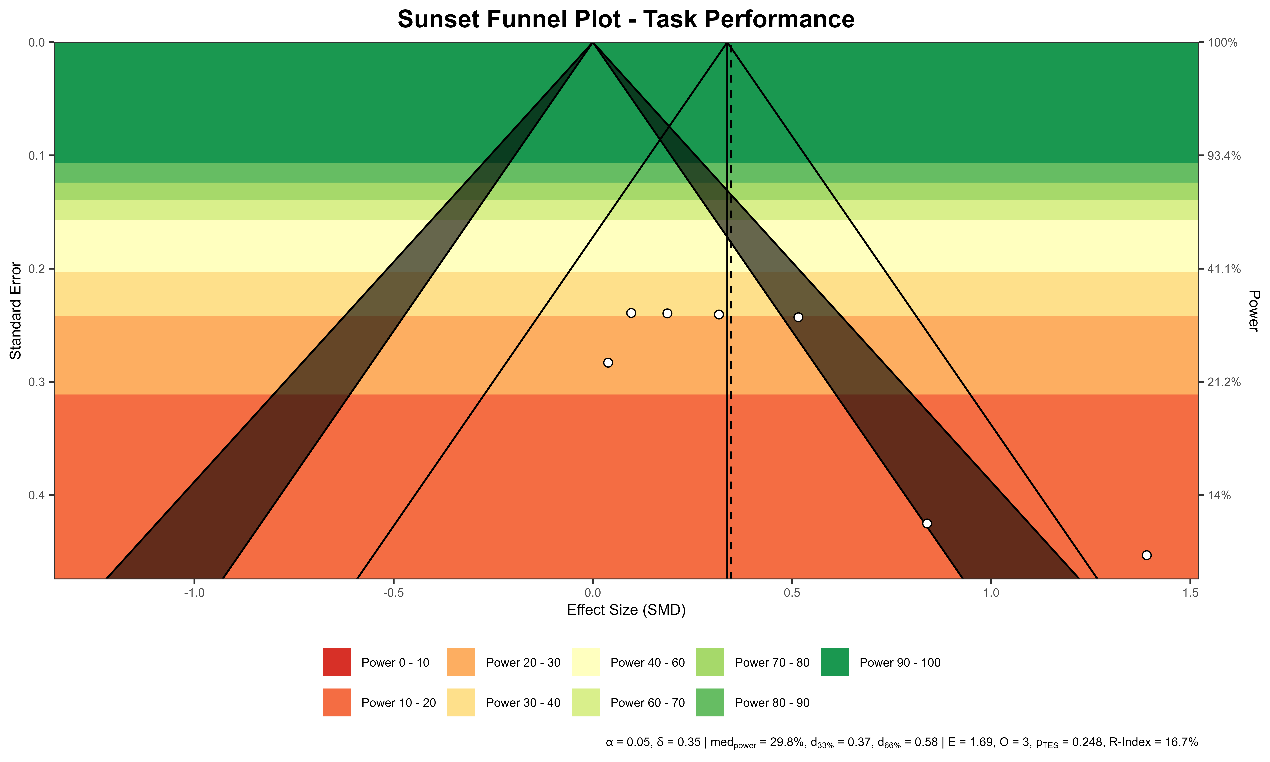


**FigureS6.** Forest plot for Task Performance (TASK)


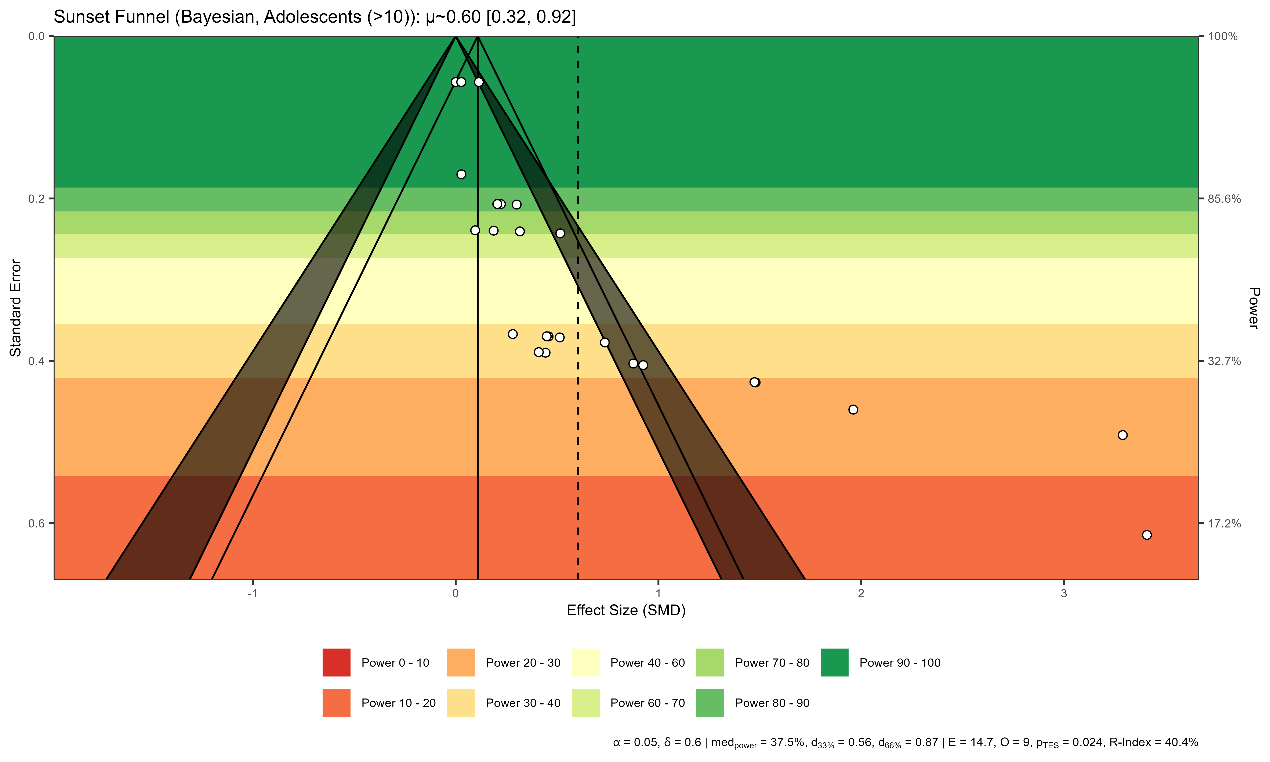


**FigureS7.** Sunset funnel plot for the Adolescents subgroup


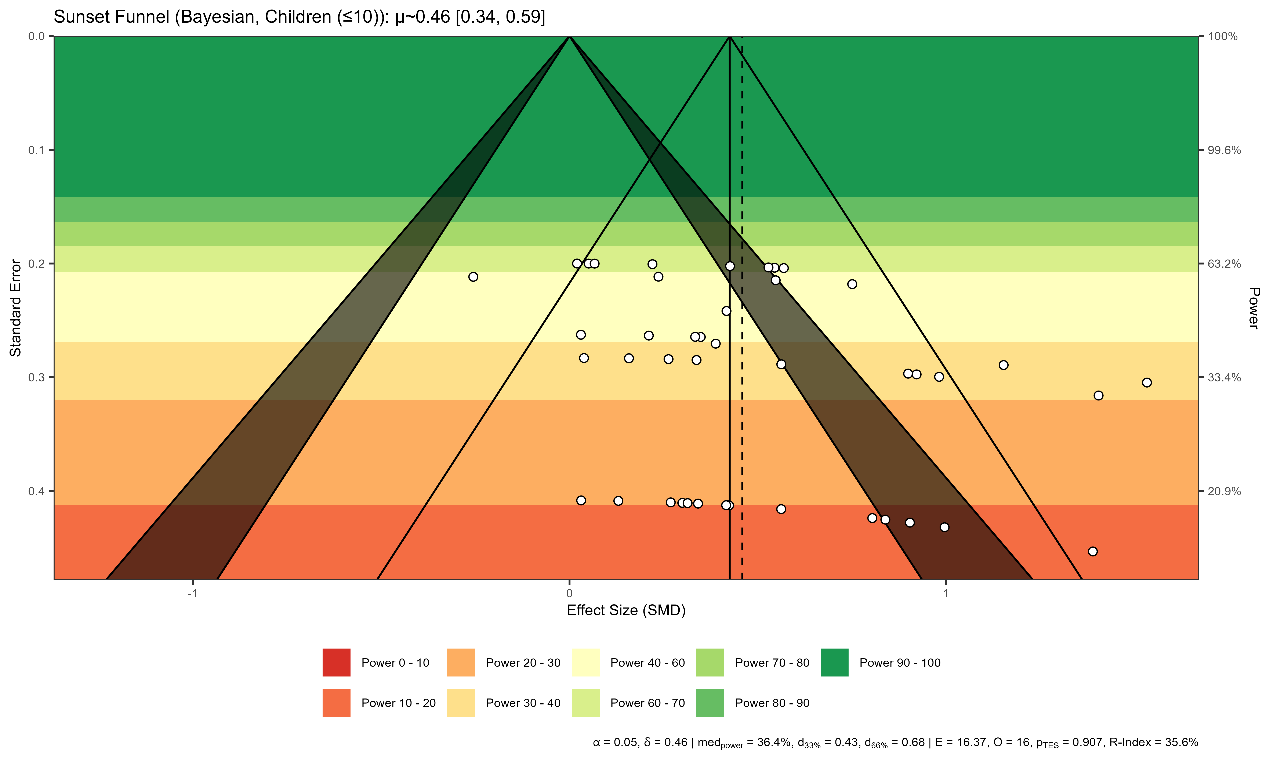


**FigureS8.** Sunset funnel plot for the Children subgroup
