# Supplementary material for: Mindfulness-based interventions for children and adolescents with attention-deficit/hyperactivity disorder: a Bayesian meta-analysis of randomized controlled trials
Source: Front Psychol. 2026 Mar 11;17:1711994. doi: 10.3389/fpsyg.2026.1711994 (PMC13013061; doi:10.3389/fpsyg.2026.1711994)
Supplement: Supplementary file 1 [file Data_Sheet_1.ZIP › supplementary file/Supplementary file S8_Full Search Strategies.pdf]

EBSCO:

TX ("mindfulness" OR "mindfulness meditation" OR "mindfulness-based" OR MBSR OR MBCT OR "mindfulness training" OR "mindfulness-based stress reduction" OR "mindfulness-based cognitive therapy") AND TX (ADHD OR "attention deficit hyperactivity disorder" OR "attention-deficit/hyperactivity disorder" OR "attention deficit disorder" OR "hyperactivity disorder" OR "hyperkinetic disorder")

Pubmed:

(

"Mindfulness"[Mesh] OR

"Mindfulness-Based Stress Reduction" OR

"Mindfulness-Based Cognitive Therapy" OR

(mindfulness OR mindful) OR

"mindfulness meditation" OR

"mindfulness-based" OR

MBSR OR MBCT OR

"mindful awareness" OR

"mindful attention" OR

"mindful practice" OR

"present-centered awareness" OR

"body scan meditation" OR --

"breath-focused meditation" OR --

"open monitoring meditation" OR -

"sitting meditation" OR

"loving-kindness meditation" --

)

AND

(

"Attention Deficit Disorder with Hyperactivity"[Mesh] OR

"Attention Deficit Disorder"[Mesh] OR

"Neurodevelopmental Disorders"[Mesh] OR

(ADHD OR ADD) OR

"attention deficit hyperactivity disorder"OR

"attention deficit disorder" OR

"hyperactivity disorder" OR

"hyperkinetic disorder" OR

"minimal brain dysfunction" OR

"executive dysfunction" OR

impulsivity OR

inattention OR

hyperactivity

)

WOS:

TS=(

("mindfulness" OR "mindful" OR "mindful awareness" OR "mindful attention" OR

"mindful practice" OR "mindfulness meditation" OR "mindfulness-based" OR

"mindful training" OR "mindful awareness practice" OR "present-centered awareness" OR

"Mindfulness-Based Stress Reduction" OR "Mindfulness Based Stress Reduction" OR

"MBSR" OR "MBSR therapy" OR "MBSR therapies" OR "Mindfulness-Based Cognitive Therapy" OR

"Mindfulness Based Cognitive Therapy" OR "MBCT" OR

"Cognitive Therapy, Mindfulness-Based" OR "Therapy, Mindfulness-Based Cognitive")

)

AND

TS=(

("Attention Deficit Hyperactivity Disorder" OR "Attention-Deficit/Hyperactivity Disorder" OR

"Attention Deficit/Hyperactivity Disorder" OR "Attention-Deficit Hyperactivity Disorder" OR

"ADHD" OR "ADD" OR

"Attention Deficit Disorder" OR "Attention Deficit Disorders" OR

"Attention Deficit Disorder with Hyperactivity" OR

"Hyperkinetic Disorder" OR "Hyperkinetic Syndrome" OR

"Childhood Hyperactivity" OR "Child Hyperactivity" OR

"Hyperactivity Disorder" OR "Hyperactivity" OR "Hyperactive Impulsive Disorder" OR

"Inattentive Disorder" OR "Impulsivity Disorder" OR "Distractibility Disorder" OR

"Minimal Brain Dysfunction" OR "Executive Dysfunction" OR

"Combined Type ADHD" OR "Predominantly Inattentive Type" OR

"Predominantly Hyperactive-Impulsive Type" OR

"Neurodevelopmental Disorder" OR "Neurodevelopmental Disorders")

)

Socpus:

TITLE-ABS-KEY(

"mindfulness" OR

"mindfulness-based" OR

"mindfulness meditation" OR

"mindfulness-based stress reduction" OR

"mindfulness based stress reduction" OR

"mbsr" OR

"mindfulness-based cognitive therapy" OR

"mindfulness based cognitive therapy" OR

"mbct" OR

"mindful awareness" OR

"mindful attention" OR

"mindful practice" OR

"mindful training" OR

"present-centered awareness" OR

"meditation-based intervention" OR

"contemplative practice" OR

"acceptance-based therapy"

)

AND

TITLE-ABS-KEY(

"attention deficit hyperactivity disorder" OR

"adhd" OR

"add" OR

"attention deficit disorder" OR

"hyperactivity disorder" OR

"hyperkinetic disorder" OR

"neurodevelopmental disorder" OR

"executive dysfunction" OR

"inattentive symptoms" OR

"hyperactivity" OR

"impulsivity" OR

"emotional dysregulation" OR

"minimal brain dysfunction" OR

"behavioral dysregulation" OR

"self-regulation difficulties"

)

Cochrane Library:

(mindfulness OR "mindfulness-based" OR "mindfulness meditation" OR "mindfulness-based stress reduction" OR "mindfulness based stress reduction" OR MBSR OR "MBSR therapy" OR "mindfulness-based cognitive therapy" OR "mindfulness based cognitive therapy" OR MBCT OR "mindful awareness" OR "mindful attention" OR "mindful practice" OR "present-centered awareness" OR "mindfulness intervention" OR "meditation-based intervention" OR "contemplative practice" OR

"acceptance-based therapy" OR "meditation" OR "meditative training")

AND

("attention deficit hyperactivity disorder" OR ADHD OR ADD OR

"attention deficit disorder" OR "hyperactivity disorder" OR

"hyperkinetic disorder" OR "childhood hyperactivity" OR

"neurodevelopmental disorder" OR "minimal brain dysfunction" OR

"executive dysfunction" OR "emotional dysregulation" OR

"behavioral dysregulation" OR "self-regulation difficulties" OR

"inattentive symptoms" OR "impulsivity" OR "self-control" OR

"executive function")

ProQuest:

TI,AB,SU(

mindfulness OR "mindfulness-based" OR

"mindfulness meditation" OR

"mindfulness-based stress reduction" OR

MBSR OR "mindfulness-based cognitive therapy" OR MBCT OR

"mindfulness intervention" OR

"meditation-based intervention" OR

"contemplative practice" OR

"acceptance-based therapy" OR "present-centered awareness"

)

AND

TI,AB,SU(

"attention deficit hyperactivity disorder" OR ADHD OR ADD OR

"attention deficit disorder" OR

"hyperactivity disorder" OR

"hyperkinetic disorder" OR

"neurodevelopmental disorder" OR

"minimal brain dysfunction" OR

impulsivity OR inattention OR

"executive dysfunction" OR

"emotional dysregulation" OR

"self-regulation difficulties"

)

ERIC:

("mindfulness" OR "mindfulness-based" OR "mindfulness meditation" OR "mindfulness-based stress reduction" OR "MBSR" OR "mindfulness-based cognitive therapy" OR "MBCT" OR "mindful attention" OR "mindful awareness" OR "mindful training" OR "present-centered awareness" OR "meditation" OR "meditation-based intervention" OR "acceptance-based therapy") AND ("attention deficit hyperactivity disorder" OR "ADHD" OR "attention deficit disorder" OR "ADD" OR "hyperactivity disorder" OR "inattention" OR "executive dysfunction" OR "behavioral problems" OR "self-regulation" OR "impulsivity" OR "neurodevelopmental disorder" OR "emotional regulation" OR "learning disabilities")

(TI("mindfulness") OR TI("mindfulness-based") OR TI("mindfulness meditation") OR TI("mindfulness-based stress reduction") OR TI("MBSR") OR TI("mindfulness-based cognitive therapy") OR TI("MBCT")

OR

AB("mindfulness") OR AB("mindfulness-based") OR AB("mindfulness meditation") OR AB("mindfulness-based stress reduction") OR AB("MBSR") OR AB("mindfulness-based cognitive therapy") OR AB("MBCT") OR AB("mindful attention") OR AB("mindful awareness") OR AB("mindful training"))

AND

(TI("attention deficit hyperactivity disorder") OR TI("ADHD") OR TI("attention deficit disorder") OR TI("ADD") OR TI("hyperactivity disorder")

OR

AB("attention deficit hyperactivity disorder") OR AB("ADHD") OR AB("attention deficit disorder") OR AB("ADD") OR AB("hyperactivity disorder"))

Embase:

(

'attention deficit disorder'/exp OR

'attention deficit hyperactivity disorder'/exp OR

'hyperkinesis'/exp OR

ADHD:ti,ab,kw OR

ADD:ti,ab,kw OR

'attention deficit*':ti,ab,kw OR

hyperkinetic*:ti,ab,kw OR

'hyperactivity disorder*':ti,ab,kw

)

AND

(

'mindfulness'/exp OR

'mindfulness based cognitive therapy'/exp OR

'mindfulness based stress reduction'/exp OR

mindfulness:ti,ab,kw OR

mindful*:ti,ab,kw OR

MBSR:ti,ab,kw OR

MBCT:ti,ab,kw OR

'mindfulness-based stress reduction':ti,ab,kw OR

'mindfulness-based cognitive therapy':ti,ab,kw OR

meditation:ti,ab,kw OR

'awareness practice*':ti,ab,kw

)
