# Supplementary material for: Mindfulness-based interventions for children and adolescents with attention-deficit/hyperactivity disorder: a Bayesian meta-analysis of randomized controlled trials
Source: Front Psychol. 2026 Mar 11;17:1711994. doi: 10.3389/fpsyg.2026.1711994 (PMC13013061; doi:10.3389/fpsyg.2026.1711994)
Supplement: Supplementary file 1 [file Data_Sheet_1.ZIP › supplementary file/Supplementary file S9_GRADE_Summary_of_Findings.pdf]

Mindfulness-based interventions for ADHD symptoms (children/adolescents)

Patient or population: Children and Adolescents With Attention-Deficit/Hyperactivity-Disorder
Settings: Outpatient, community, and school settings
Intervention: mindfulness-based interventions

Outcomes	Illustrative comparative risks* (95% CI)		Relative effect (95% CI)	No of Participants (studies)	Quality of the evidence (GRADE)	Comments
	Assumed risk	Corresponding risk				
	Control	Mindfulness-based interventions				
**Executive Function**<br>Follow-up: median 8 weeks		The mean executive function in the intervention groups was **0.31 standard deviations higher** (0.26 lower to 0.89 higher)		2694 (14 studies)	⊕⊕⊕⊖ **moderate**[1]	
**Inattention**<br>Follow-up: median 8 weeks		The mean inattention in the intervention groups was **0.37 standard deviations higher** (0.13 lower to 0.87 higher)		3269 (18 studies)	⊕⊕⊕⊖ **moderate**[2]	
**Hyperactivity/Impulsivity**<br>Follow-up: median 8 weeks		The mean hyperactivity/impulsivity in the intervention groups was **0.61 standard deviations higher** (0.32 to 0.9 higher)		2525 (16 studies)	⊕⊕⊕⊖ **moderate**[2]	
**Task Performance**<br>Follow-up: median 8 weeks		The mean task performance in the intervention groups was **0.44 standard deviations higher** (0.07 lower to 0.95 higher)		145 (7 studies)	⊕⊕⊕⊖ **moderate**[2]	
**Global**<br>Follow-up: median 8 weeks		The mean global in the intervention groups was **1.44 standard deviations higher** (0.81 to 2.06 higher)		324 (11 studies)	⊕⊕⊕⊖ **moderate**[2]	
**Emotional**<br>Follow-up: median 8 weeks		The mean emotional in the intervention groups was **0.41 standard deviations higher** (0.09 to 0.74 higher)		145 (3 studies)	⊕⊕⊕⊖ **moderate**[2]	

*The basis for the **assumed risk** (e.g. the median control group risk across studies) is provided in footnotes. The **corresponding risk** (and its 95% confidence interval) is based on the assumed risk in the comparison group and the **relative effect** of the intervention (and its 95% CI).

CI: Confidence interval;

GRADE Working Group grades of evidence
High quality: Further research is very unlikely to change our confidence in the estimate of effect.
Moderate quality: Further research is likely to have an important impact on our confidence in the estimate of effect and may change the estimate.
Low quality: Further research is very likely to have an important impact on our confidence in the estimate of effect and is likely to change the estimate.
Very low quality: We are very uncertain about the estimate.

[1] participant/personnel blinding not reported across trials; EF largely rated outcomes susceptible to performance bias
[2] No explanation was provided

Author(s): Yi Ran Liu, MSc, Macau Polytechnic University, Macao SAR, China
Date: 2025-07-01
Question: Should mindfulness-based interventions be used in Children and Adolescents With Attention-Deficit/Hyperactivity-Disorder?
Settings: Outpatient, community, and school settings
Bibliography: Liu YR, et al. Mindfulness-based interventions for ADHD symptoms in children and adolescents: a systematic review and meta-analysis. Unpublished manuscript, 2025.

Quality assessment							No of patients		Effect		Quality	Importance
No of studies	Design	Risk of bias	Inconsistency	Indirectness	Imprecision	Other considerations	Mindfulness-based interventions	Control	Relative (95% CI)	Absolute		
**Executive Function (follow-up median 8 weeks; Better indicated by lower values)**												
14	randomised trials	serious[1]	no serious inconsistency	no serious indirectness	no serious imprecision	none	1985	709	-	SMD 0.31 higher (0.26 lower to 0.89 higher)	⊕⊕⊕O MODERATE	CRITICAL
**Inattention (follow-up median 8 weeks; Better indicated by higher values)**												
18	randomised trials	serious[2]	no serious inconsistency	no serious indirectness	no serious imprecision	none	2271	998	-	SMD 0.37 higher (0.13 lower to 0.87 higher)	⊕⊕⊕O MODERATE	CRITICAL
**Hyperactivity/Impulsivity (follow-up median 8 weeks; Better indicated by higher values)**												
16	randomised trials	serious[2]	no serious inconsistency	no serious indirectness	no serious imprecision	none	1896	629	-	SMD 0.61 higher (0.32 to 0.9 higher)	⊕⊕⊕O MODERATE	CRITICAL
**Task Performance (follow-up median 8 weeks; Better indicated by higher values)**												
7	randomised trials	serious[2]	no serious inconsistency	no serious indirectness	no serious imprecision	none	76	69	-	SMD 0.44 higher (0.07 lower to 0.95 higher)	⊕⊕⊕O MODERATE	IMPORTANT
**Global (follow-up median 8 weeks; Better indicated by higher values)**												
11	randomised trials	serious[2]	no serious inconsistency	no serious indirectness	no serious imprecision	none	164	160	-	SMD 1.44 higher (0.81 to 2.06 higher)	⊕⊕⊕O MODERATE	CRITICAL
**Emotional (follow-up median 8 weeks; Better indicated by higher values)**												
3	randomised trials	serious[2]	no serious inconsistency	no serious indirectness	no serious imprecision	none[2]	74	71	-	SMD 0.41 higher (0.09 to 0.74 higher)	⊕⊕⊕O MODERATE	IMPORTANT

[1] participant/personnel blinding not reported across trials; EF largely rated outcomes susceptible to performance bias
[2] No explanation was provided
